# Evidence for a Diagenetic Origin of Vera Rubin Ridge, Gale Crater, Mars: Summary and Synthesis of *Curiosity*'s Exploration Campaign

**DOI:** 10.1029/2020JE006527

**Published:** 2020-12-23

**Authors:** A. A. Fraeman, L. A. Edgar, E. B. Rampe, L. M. Thompson, J. Frydenvang, C. M. Fedo, J. G. Catalano, W. E. Dietrich, T. S. J. Gabriel, A. R. Vasavada, J. P. Grotzinger, J. L'Haridon, N. Mangold, V. Z. Sun, C. H. House, A. B. Bryk, C. Hardgrove, S. Czarnecki, K. M. Stack, R. V. Morris, R. E. Arvidson, S. G. Banham, K. A. Bennett, J. C. Bridges, C. S. Edwards, W. W. Fischer, V. K. Fox, S. Gupta, B. H. N. Horgan, S. R. Jacob, J. R. Johnson, S. S. Johnson, D. M. Rubin, M. R. Salvatore, S. P. Schwenzer, K. L. Siebach, N. T. Stein, S. M. R. Turner, D. F. Wellington, R. C. Wiens, A. J. Williams, G. David, G. M. Wong

**Affiliations:** ^1^ Jet Propulsion Laboratory California Institute of Technology Pasadena CA USA; ^2^ U.S. Geological Survey Astrogeology Science Center Flagstaff AZ USA; ^3^ NASA Johnson Space Center Houston TX USA; ^4^ Planetary and Space Science Centre University of New Brunswick Fredericton New Brunswick Canada; ^5^ Global Institute University of Copenhagen Copenhagen Denmark; ^6^ Department of Earth and Planetary Sciences University of Tennessee, Knoxville Knoxville TN USA; ^7^ Department of Earth and Planetary Sciences Washington University in St. Louis St. Louis MO USA; ^8^ Department of Earth and Planetary Science University of California Berkeley CA USA; ^9^ School of Earth and Space Exploration Arizona State University Tempe AZ USA; ^10^ Division of Geological and Planetary Sciences California Institute of Technology Pasadena CA USA; ^11^ Laboratoire de Planétologie et Géodynamique de Nantes, UMR6112 CNRS Université de Nantes, Université d'Angers Nantes France; ^12^ Department of Geosciences Pennsylvania State University University Park PA USA; ^13^ Department of Earth Science and Engineering Imperial College London London UK; ^14^ Space Research Centre, School of Physics and Astronomy University of Leicester Leicester UK; ^15^ Department of Astronomy and Planetary Science Northern Arizona University Flagstaff AZ USA; ^16^ Department of Earth Sciences University of Minnesota, Twin Cities Minneapolis MN USA; ^17^ Department of Earth, Atmospheric, and Planetary Sciences Purdue University West Lafayette IN USA; ^18^ Johns Hopkins University Applied Physics Laboratory Laurel MD USA; ^19^ Department of Biology, Science, Technology, and International Affairs Program Georgetown University Washington DC USA; ^20^ Department of Earth and Planetary Sciences University of California Santa Cruz CA USA; ^21^ AstrobiologyOU The Open University Milton Keynes UK; ^22^ Department of Earth, Environmental, and Planetary Sciences Rice University Houston TX USA; ^23^ Los Alamos National Laboratory Los Alamos NM USA; ^24^ Department of Geological Sciences University of Florida Gainesville FL USA; ^25^ L'Institut de Recherche en Astrophysique et Planétologie Toulouse France

**Keywords:** Mars, Diagenesis, Hematite, Lacustrine, Curiosity

## Abstract

This paper provides an overview of the *Curiosity* rover's exploration at Vera Rubin ridge (VRR) and summarizes the science results. VRR is a distinct geomorphic feature on lower Aeolis Mons (informally known as Mount Sharp) that was identified in orbital data based on its distinct texture, topographic expression, and association with a hematite spectral signature. *Curiosity* conducted extensive remote sensing observations, acquired data on dozens of contact science targets, and drilled three outcrop samples from the ridge, as well as one outcrop sample immediately below the ridge. Our observations indicate that strata composing VRR were deposited in a predominantly lacustrine setting and are part of the Murray formation. The rocks within the ridge are chemically in family with underlying Murray formation strata. Red hematite is dispersed throughout much of the VRR bedrock, and this is the source of the orbital spectral detection. Gray hematite is also present in isolated, gray‐colored patches concentrated toward the upper elevations of VRR, and these gray patches also contain small, dark Fe‐rich nodules. We propose that VRR formed when diagenetic event(s) preferentially hardened rocks, which were subsequently eroded into a ridge by wind. Diagenesis also led to enhanced crystallization and/or cementation that deepened the ferric‐related spectral absorptions on the ridge, which helped make them readily distinguishable from orbit. Results add to existing evidence of protracted aqueous environments at Gale crater and give new insight into how diagenesis shaped Mars' rock record.

## Key Points

- We summarize *Curiosity*'s campaign at Vera Rubin ridge (Sols 1726–2302) and the high‐level results from articles in this special issue
- Vera Rubin ridge formed when diagenesis hardened rocks along the base of Aeolis Mons; wind subsequently etched the feature into a ridge
- Results add evidence for protracted aqueous environments at Gale crater and give new insight into how diagenesis shaped Mars' rock record

## 1 Introduction

**Figure 1:**
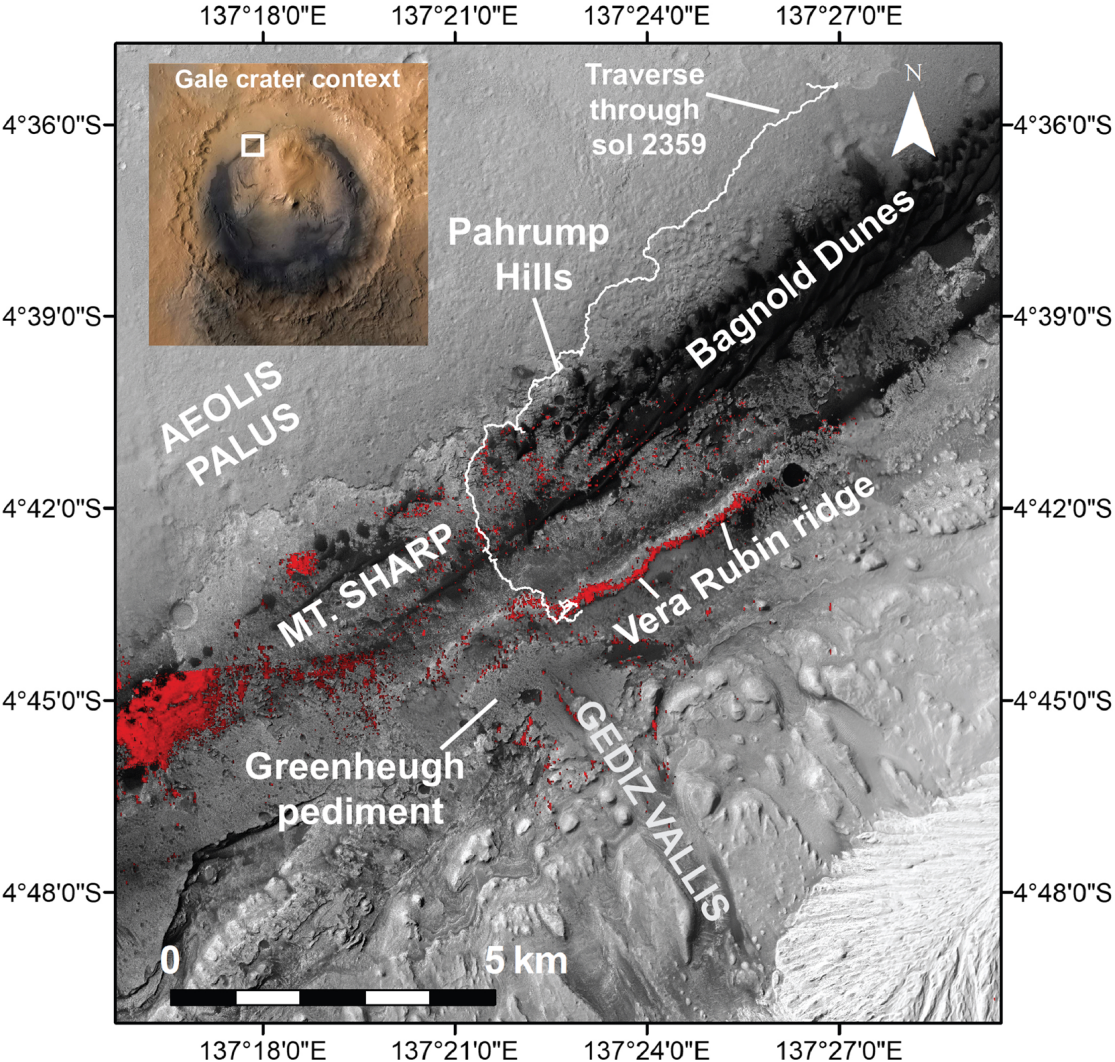
Overview of *Curiosity*'s traverse through Sol 2359 (white line) over mosaic of High Resolution Imaging Science Experiment (HiRISE) images showing the northwest quadrant of Mount Sharp. Key named features are indicated, and a zoomed out context image showing all of Gale crater is shown in the upper left. Red areas show locations with 860 nm absorption in Compact Reconnaissance Imaging Spectrometer for Mars (CRISM) data, which are interpreted to indicate the presence of red crystalline hematite (from Fraeman et al., 2016).

The Mars Science Laboratory rover *Curiosity* is investigating Mars' habitability by documenting ancient environments that are preserved in the planet's sedimentary rock record (Grotzinger et al., 2012). *Curiosity* landed on the floor of the ~155 km diameter Gale crater in August 2012 and began a traverse that ultimately led to Aeolis Mons, a ~5 km tall mound of sedimentary rock informally called Mount Sharp (Figure 1). Mount Sharp's strata record evidence of several unique, and potentially globally significant, environmental changes within an established stratigraphic context (Anderson & Bell, 2010; Golombek et al., 2012; Milliken, Grotzinger, & Thomson, 2010). Since reaching the base of Mount Sharp in 2014, *Curiosity* has climbed over 370 m in elevation and found evidence of lacustrine and lacustrine margin settings (Grotzinger et al., 2015; Stack et al., 2019) in which organic materials are preserved (Eigenbrode et al., 2018; Freissinet et al., 2015).

**Figure 2:**
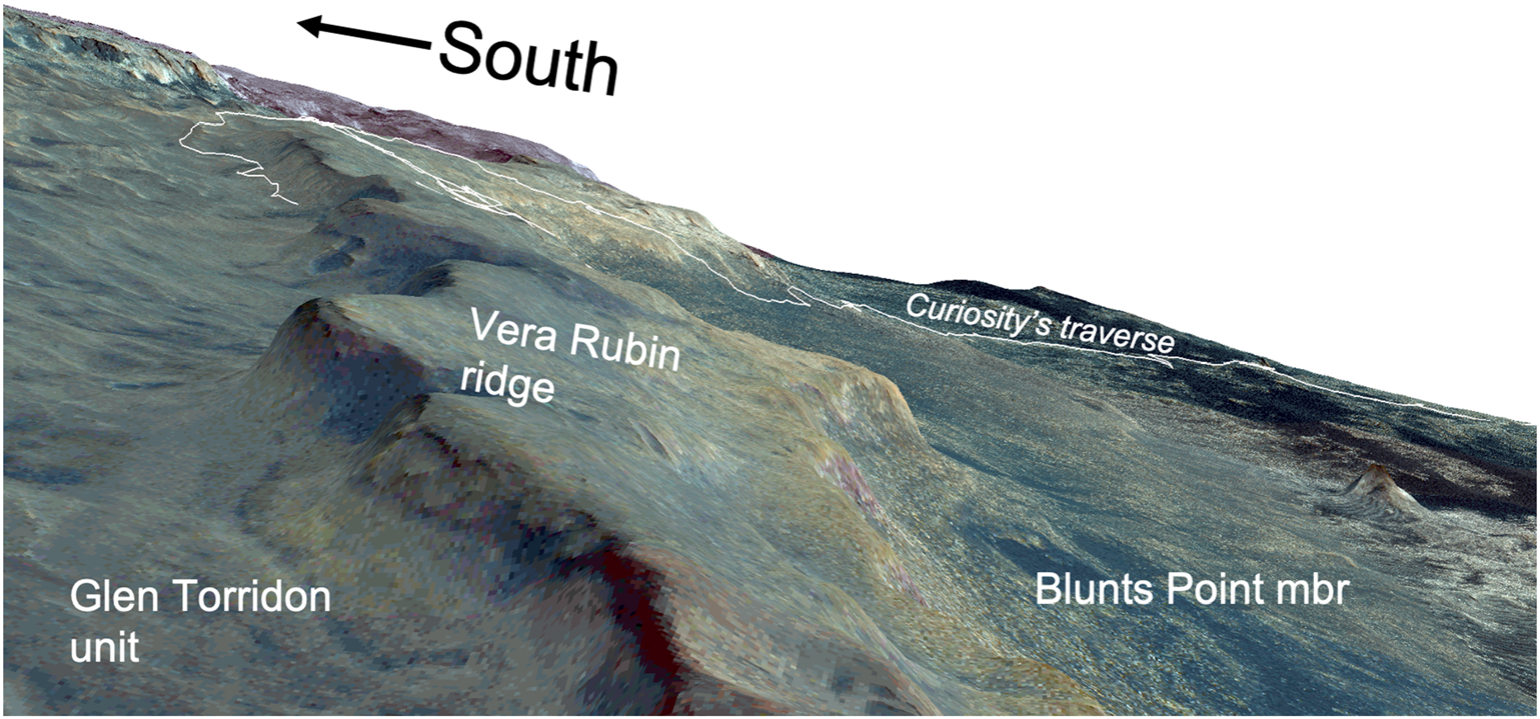
Color image from the High Resolution Imaging Science Experiment (HiRISE) camera draped on 2 times vertically exaggerated HiRISE stereo digital elevation model that shows a perspective view of Vera Rubin ridge. Image is looking along the ridge toward the southeast, and *Curiosity*'s traverse through Sol 2359 is shown as a white line.

In September 2017 *Curiosity* ascended a layered ridge on the northwest flank of Mount Sharp (Figures 1 and 2). This feature was called Vera Rubin ridge (VRR) to honor the pioneering American astronomer Vera Cooper Rubin (1928–2016). Dr. Rubin's precise measurements of the rotation rates of galaxies revealed the existence of dark matter. She was also a fierce advocate for the equal treatment of women in science (Bahcall, 2017).

VRR is one of several geomorphic features in Mount Sharp that had been recognized in orbital images and spectroscopic data before the rover's arrival in Gale crater (Anderson & Bell, 2010; Fraeman et al., 2016; Milliken et al., 2010; Thomson et al., 2011). In addition to being distinguishable by its elevated topography (Figure 2), the ridge is associated with strong spectral absorptions that are attributed to crystalline red hematite in data from the Compact Reconnaissance Imaging Spectrometer for Mars (CRISM) (Fraeman et al., 2013) (Figure 1). Red hematite is defined by its red color in visible light and is finer grained (~10–100 nm up to <3–5 μm grain sizes) than gray hematite, which appears gray to black in visible light (> ~3–5 μm grain sizes) (Catling & Moore, 2003; Morris et al., 2020; Sherman & Waite, 1985).

Based on the orbital spectral observations, VRR was originally interpreted to be an isolated hematite‐bearing sedimentary interval within Mount Sharp, and two hypotheses were proposed to explain the formation of this apparently localized hematite deposit (Fraeman et al., 2013). In the first hypothesis, soluble Fe^2+^ ions were carried in solution by anoxic fluids until they encountered an oxidizing environment, at which point insoluble Fe^3+^ minerals precipitated. Geologic settings where this could occur include precipitation in a subaqueous environment at a redox interface between anoxic and oxidizing waters (e.g., redox‐stratified lake; Hurowitz et al., 2017) or precipitation from anoxic groundwater exposed to an oxidizing subaqueous or subaerial environment (i.e., shoreline deposit or spring deposit). The second hypothesis was that the ridge area experienced local in‐place oxidative weathering of Fe^2+^ minerals. This could occur via oxidative weathering by near‐neutral pH waters (e.g., red beds; Walker, 1967) or acidic waters or vapor (e.g., Mauna Kea tephras; Graff et al., 2014). A third hypothesis, open‐system oxidative weathering resulting in a lag of insoluble phases that included ferric phases, was deemed less likely based on the dearth of evidence in orbital data for other associated mineral phases that would be expected in this environment, including aluminous clays and silica‐rich phases (e.g., laterite).

The preferred two hypotheses both explain the apparent concentration of hematite in VRR observed by CRISM suggested this location was a site of past iron oxidation. Abiotic processes can oxidize iron in aqueous environments on Mars via chemical reaction with O_2_, H_2_O_2_, or chlorate, or by photooxidation (Brundrett et al., 2019; Hurowitz et al., 2010; Mitra & Catalano, 2019; Nie et al., 2017). However, on Earth, oxidation and reduction of iron at redox gradients is often catalyzed by microbes, thus linking VRR with a possible habitable setting (Allen et al., 2001; Hays et al., 2017).

Once *Curiosity* reached Mount Sharp, evidence for early and late diagenesis (i.e., physical and chemical changes to sediments after deposition) was pervasive in the form of spherules of different compositions, mineralized veins, and lenticular crystal molds (e.g., Hurowitz et al., 2017; Kah et al., 2018; Kronyak et al., 2019; Siebach et al., 2014; Sun et al., 2019). Additionally, data from *Curiosity*'s instruments revealed abundant hematite and Fe^3+^‐bearing clay minerals in Mount Sharp stratigraphically below VRR. Seven of the nine samples drilled from Murray formation sedimentary rocks leading up to the ridge contained between ~2 and ~12 wt % hematite (Bristow et al., 2018; Rampe et al., 2017). Furthermore, in hundreds of meters of section that *Curiosity* did not drill, spectral data indicated ferric oxides were present within the predominantly lacustrine mudstone (Fraeman et al., 2020 *;* Johnson et al., 2016; Wellington et al., 2017). Hypotheses for hematite formation here included precipitation in shallow, oxic lake waters (Hurowitz et al., 2017) or crystallization from a precursor through diagenesis by groundwater (Rampe et al., 2017).

The discovery of hematite in Mount Sharp stratigraphically below VRR suggested that the ridge was not uniquely hematite‐bearing, so the key question concerning VRR evolved from “Why is there a distinct location in orbital data over Mount Sharp that has hematite?” to “How does the hematite in VRR relate to units stratigraphically below?” and “Why is the spectral signature of hematite so strong at VRR in orbital data?” Additionally, textural evidence of pervasive diagenesis along with the model that hematite below VRR formed by diagenesis from groundwater (Rampe et al., 2017) led to a new hypothesis not originally considered from orbital data alone, that early or late diagenesis by oxic fluids was a potential formation mechanism for VRR.

*Curiosity* spent more than an Earth year exploring VRR, collecting detailed textural, sedimentological, and compositional information. These data give insight into the origin and evolution of both the ridge‐forming strata and the ridge itself, the ridge's relationship with the surrounding terrain, and the source of the CRISM hematite signature. Here we provide an overview of the design and implementation of *Curiosity*'s scientific campaign at the ridge and then synthesize the high‐level science results.

## 2 Geologic Setting

VRR is a ~6.5 km long northeast‐southwest trending ridge that is ~200 m wide (Figures 1 and 2). Consistent with the northwest regional slope of Mount Sharp, the upslope (southern) edge of the ridge is higher than the downslope (northern) side. The top of the ridge in the north to the top of the ridge in the south spans ~50 m of elevation, and the northern facing slope of the ridge rises ~100 m above the sloping basal plains below. There is heterogeneity in the color and textures of VRR viewed from orbit. The upper portion of the ridge is darker and more heavily cratered than the lower portion of the ridge. VRR also has a higher thermal inertia compared with surrounding terrain in orbital data sets, ~350–400 J m^−2^ K^−1^ s^−1/2^ on the ridge versus ~200–250 J m^−2^ K^−1^ s^−1/2^ in the surroundings (Edwards et al., 2018).

**Figure 3:**
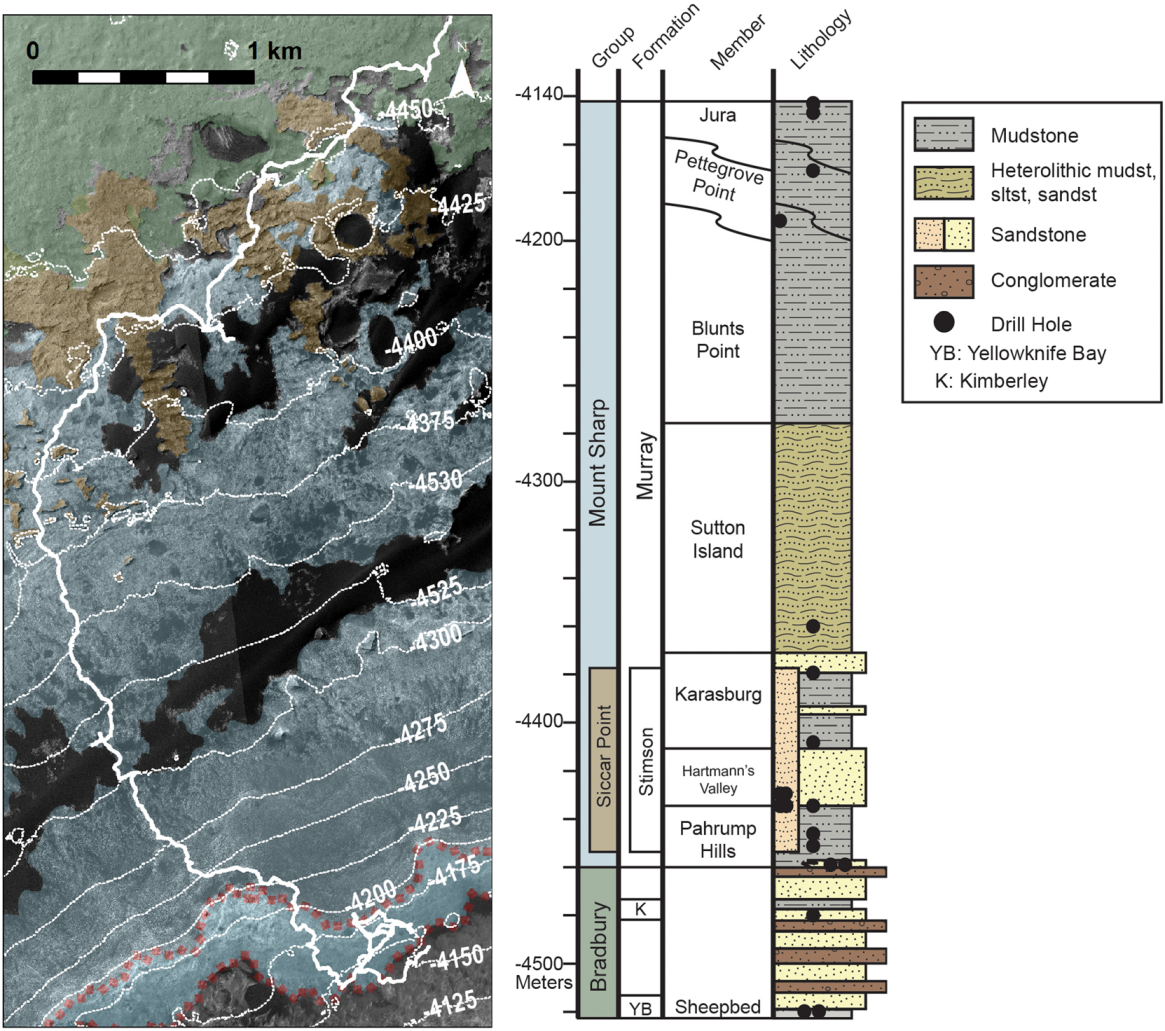
(left) *Curiosity*'s traverse on Mount Sharp through Sol 2359 with 25 m contours. Stratigraphic groups defined from *Curiosity* data and extrapolated to orbital view are shown. Bradbury group is mapped in green, Mount Sharp group in blue, and Siccar Point group in brown. The extent of the original Vera Rubin ridge geomorphic unit is shown outlined in red, and *Curiosity* data showed rocks within the ridge were part of the Murray formation within the Mount Sharp group. (right) Associated stratigraphic column along *Curiosity*'s traverse from Edgar et al. (2020). VRR is composed of the Pettegrove Point and Jura members.

Analysis of Mount Sharp strata below VRR via *Curiosity* observations shows that they were predominantly deposited in a lacustrine setting (Fedo et al., 2019; Grotzinger et al., 2015; Gwizd et al., 2019; Stack et al., 2019). These strata are defined as the Murray formation, which is the only major formation explored by *Curiosity* to date within the Mount Sharp group, and are subdivided into five lithostratigraphic members below VRR (Fedo et al., 2019). In order of increasing elevation, the members are the “Pahrump Hills” member, “Hartmann's Valley” member, “Karasburg” member, “Sutton Island” member, and “Blunts Point” member (Figure 3). Pahrump Hills and parts of the Karasburg member are composed of persistent finely laminated mudstones. Hartmann's Valley and other parts of the Karasburg member contain mudstones and sandstones with decimeter‐ to meter‐scale cross bedding. The Blunts Point member, which sits directly below VRR strata, is a heterolithic assemblage including mudstones, siltstones, and sandstones.

**Figure 4:**
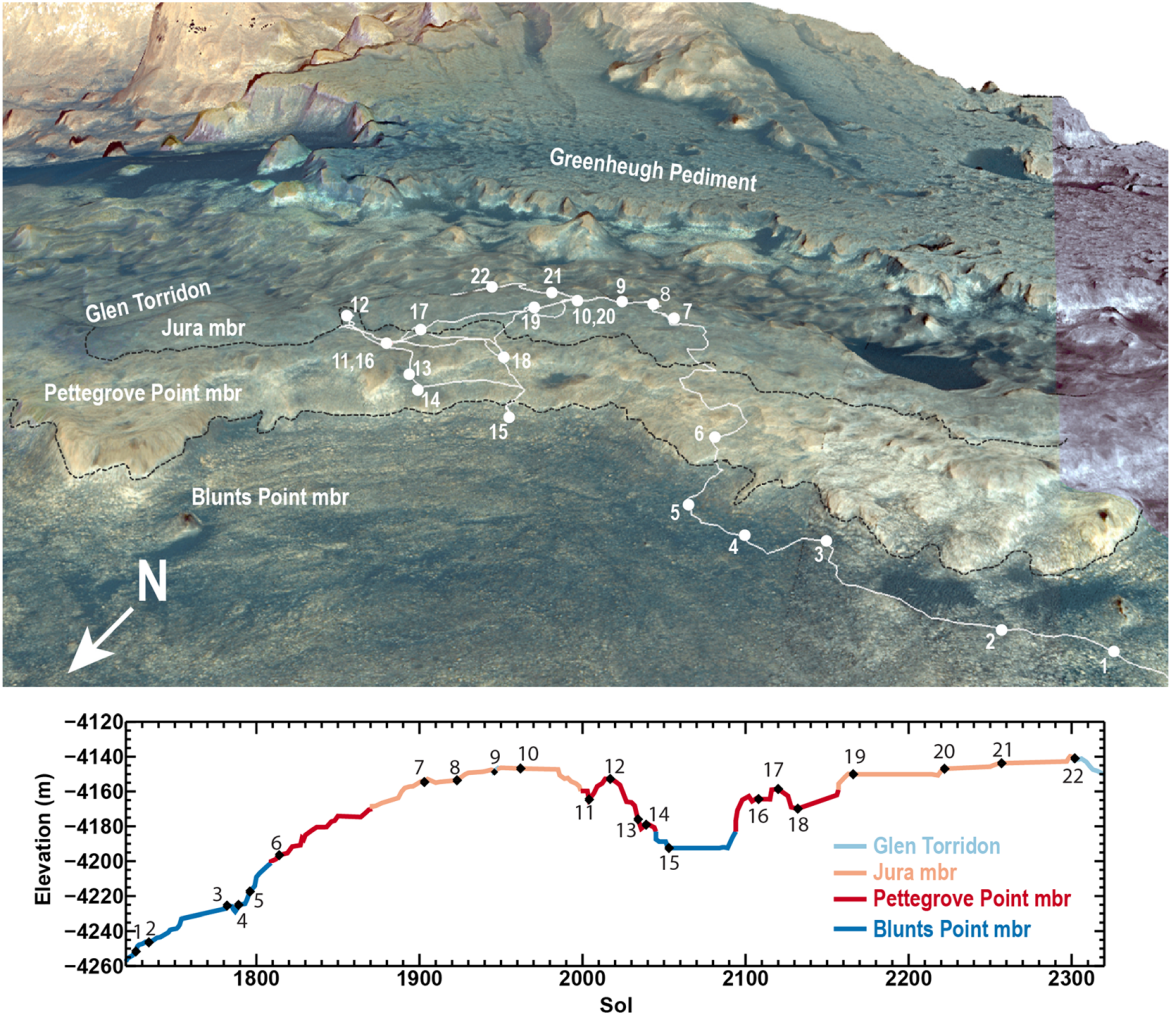
(top) Perspective view of *Curiosity*'s traverse over VRR with stratigraphic members and key campaign stops labeled. (bottom) Sol versus elevation along traverse. Numbered stops key: (1–5) approach imaging stops, (6) fracture investigation, (7, 8) first gray patch investigation, (9) toe dip into Glen Torridon unit, (10) Lake Orcadie rotary‐only drill attempt, (11) reconnaissance of area with strongest CRISM spectral signature, (12) Bressay deposit investigation, (13) Taconite crater, (14) Red Cliff imaging stop, (15) Blunts Point drill, (16) Voyageurs failed drill, (17) Ailsa Craig failed drill, (18) Stoer drill, (19) Inverness failed drill, (20) Highfield drill, (21) Rock Hall drill, and (22) transition to Glen Torridon.

The ridge occurs downslope of Gediz Vallis and the Greenheugh pediment (Figures 1 and 4). Gediz Vallis is a ~9.5 km long trough that extends southward down Mount Sharp. The capping rocks of the Greenheugh pediment sit at the base of Gediz Vallis and form part of the Siccar Point group, which rests unconformably on Mount Sharp group sedimentary rocks (Anderson & Bell, 2010; Banham et al., 2018; Bryk et al., 2019; Fraeman et al., 2016; Grotzinger et al., 2015). Topographic projections demonstrate that the surface exposures on VRR that *Curiosity* visited would have been covered by the pediment‐capping unit if the unit had once extended farther north (Bryk et al., 2019). The ridge is slightly sinuous, with the largest direction deviation opposite the Greenheugh pediment.

CRISM data show that VRR is associated with spectra that have absorptions at 530 and 860 nm, as a well a local reflection maximum near 750 nm, which are diagnostic of red crystalline hematite (Fraeman et al., 2013). Several other locations in lower Mount Sharp also have spatially coherent, strong spectral absorptions that are also consistent with hematite (Fraeman et al., 2016; Milliken et al., 2010), but VRR is unique among these detections because the deep spectral absorptions attributed to hematite clearly align with a morphologic feature (Figure 1).

## 3 Campaign Goals and Objectives

The overarching aims of *Curiosity*'s campaign at VRR were to reconstruct the past Martian environments that are preserved in the ridge's strata and to determine whether these environments could have been habitable. Three campaign‐level goals were developed based on orbital data analyses before *Curiosity* reached the ridge. These goals guided strategic route planning activities and established the key measurements to be made using *Curiosity*'s payload instruments (Table 1).

**Table 1:** The Primary Instruments Used to Address VRR Campaign Goals

Instrument name	Abbreviation	Data returned	Instrument reference
Alpha‐Particle X‐Ray Spectrometer	APXS	Elemental chemistry integrated over ~15 mm −30 mm diameter area	Gellert and Clark (2015)
Chemistry and Mineralogy	CheMin	Bulk mineralogy from powdered drill sample	Blake et al. (2012)
Chemistry and Camera	ChemCam	Elemental chemistry from ~350–550 μm areas; visible spectral from 1.3–4.5 mm areas; high‐resolution images	Johnson et al. (2015); Le Mouélic et al. (2015); Maurice et al. (2012); Maurice et al. (2016); Wiens et al. (2012)
Dynamic Albedo of Neutrons	DAN	Abundance of subsurface neutron scatterers (H) and absorbers (e.g., Cl, Fe)	Mitrofanov et al. (2012); Sanin et al. (2015)
Mars Hand Lens Imager	MAHLI	Close up color images with resolution dependent on standoff distance; ~17 μm/pixel at typical 3 cm standoff	Edgett et al. (2012)
Mast Cameras	Mastcam	Landscape color and 13‐band multispectral images with resolution of 450 μm/pixel (M34 camera) and 150 μm/pixel (M100 camera) for targets 2 meters away	Bell et al. (2017); Malin et al. (2017)
Sample Analysis at Mars	SAM	Volatiles, organics, and isotopic compositions from a powdered drill sample	Mahaffy et al. (2012)

### 3.1 *Campaign Goal 1:* Understand the Primary Depositional Setting of the Sedimentary Rocks That Make Up the Ridge and Document Their Stratigraphic Relationship With Surrounding Units

Characterizing the depositional setting(s) of VRR was critical for constraining past Martian conditions and placing *Curiosity*'s mineralogical and geochemical measurements in context. VRR appears stratified in 25 cm/pixel High Resolution Imaging Science Experiment (HiRISE) images. Although layered rocks seen from orbit could be lava flows, the lack of nearby volcanic vents, fissures, or other obvious evidence of volcanism in the area favored a sedimentary hypothesis for rocks that compose the ridge. Orbital data were not sufficient to conclude whether the sedimentary environment was lacustrine, fluvial, or eolian.

Understanding the stratigraphic relationship between the rocks of VRR and surrounding units was necessary to constrain when the feature formed with respect to Mount Sharp. One key question was whether the rocks exposed on VRR form part of the Mount Sharp sedimentary sequence or whether they represented a younger unconformable unit. Digital elevation models (DEMs) constructed using stereo images acquired by the HiRISE camera (1 m/post) were consistent with an interpretation that the dips of ridge layers were in family with similarly measured stratal dips of the overlying sulfate‐rich unit (Fraeman et al., 2013). This observation supports the idea that the rocks composing the ridge were part of the primary Mount Sharp sedimentary sequence. However, VRR's stratigraphic relationship with sedimentary rocks exposed in the trough immediately to the south, a region informally named Glen Torridon, could not be uniquely constrained with data collected from orbit (Stein et al., 2020).

Three measurement objectives were developed to support this goal: (1) acquire images of the base of VRR and study them for any evidence of gaps in the stratigraphic record or evidence for depositional hiatuses; (2) take stereo images bedding within VRR bedrock to obtain measurements of stratal strike and dip; and (3) collect high‐resolution, close‐up imaging of dust‐free surfaces to characterize grain size, sorting, and grain roundness within VRR bedrock. All three objectives were achieved using the ChemCam RMI, Mastcam, and MAHLI instruments.

### 3.2 *Campaign Goal 2:* Determine the Source of the Orbital Hematite Signature, Understand Its Relationship With Other Hematite Detections in Mount Sharp, and Test the Hypothesis That the Hematite Associated With the Ridge Indicated a Site of Past Iron Oxidation

*Curiosity* discovered hematite in samples drilled from Mount Sharp bedrock below VRR (Bristow et al., 2018; Rampe et al., 2017). Rampe et al. (2017) proposed that hematite formed during multiple influxes of mildly acidic and oxidizing diagenetic fluids. Hurowitz et al. (2017) alternatively suggested that ferric phases precipitated directly in an oxic‐anoxic mixing zone in a redox‐stratified, neutral‐alkaline lake. Dioctahedral smectites (Al, Fe^3+^) were also observed in association with hematite, and these were proposed to indicate open‐system alteration of basaltic sediments with oxidation driven by periodic desiccation and migration of the water table (Bristow et al., 2018). Determining how, and if, hematite in the ridge was related to the detections of oxidized phases in the underlying strata was a significant question on approach to VRR. A related question was whether hematite evolved from primary ferric precipitates, if it is connected to iron oxidation during a late diagenetic event, or both occurred.

In CRISM data, hematite‐related spectral absorptions along *Curiosity*'s traverse up Mount Sharp are much weaker than those in VRR (Figure 1) (Fraeman et al., 2016; Milliken et al., 2010). *Curiosity* similarly observed an enhanced hematite spectral signature associated with VRR compared with underlying strata using the Mastcam multispectral imager and ChemCam in passive spectral mode, from kilometers away (Johnson et al., 2016; Wellington et al., 2017). Hypotheses for the source of VRR's unique spectral properties were either that it contained a greater total abundance of hematite or that the combination of number of pigmenting hematite particles, hematite grain size variations, and associated phases had a stronger effect on spectral properties (i.e., Lane et al., 2002; Morris et al., 1989; Morris et al., 2020). Combined, the tasks of determining the source of the orbital hematite signature and how it linked to previous detections would address the original hypothesis that VRR was a uniquely hematite‐bearing layer that represented a site of localized iron oxidation.

Three measurement objectives were developed to support this goal: (1) obtain chemical, mineralogical, and spectral reflectance measurements from the area where CRISM and long‐distance in situ spectral data showed the deepest hematite‐related spectral signatures; (2) document variations in spectral properties, chemistry, and mineralogy at numerous locations across the ridge; and (3) acquire periodic multispectral images of the terrain to search for evidence of ferric phases following or crosscutting stratigraphy. These objectives were achieved using the full suite of *Curiosity* science instruments in Table 1.

### 3.3 *Campaign Goal 3:* Document Additional Primary and Secondary Geochemical Environments That Shaped the Ridge

It was known in advance of *Curiosity*'s investigation of VRR that there were almost certainly minerals in VRR that are not visible to orbital instruments because of the instruments' spatial resolution and depth of sensing limitations, as well as knowledge that many minerals do not have diagnostic absorptions in CRISM wavelength's range. However, detecting and characterizing all as many components as possible within VRR by *Curiosity* is important for constraining the full range of primary and secondary environments preserved within the ridge. Of particular interest is evidence for a cementing phase(s) that led to the relative erosional resistance of the ridge. If a cement is present, what is its composition? Alternatively, small variations in grain size linked to depositional changes could have caused minor differences in rock strength, and these strength differences would have been emphasized by billions of years of erosion by the Martian wind.

Measurement objectives needed to address this goal were similar to the objectives for Goal 2. They included collecting systematic detailed textural, chemical, and mineralogical data in order to document the full diversity of rocks within VRR.

## 4 Brief Overview of *Curiosity*'s Activities at VRR

The scientific goals presented in section 3 established the strategic planning framework for *Curiosity*'s VRR campaign, and activities were also modified at times to respond to in situ discoveries and rover technical issues. A detailed breakdown of *Curiosity*'s activities on VRR is shown in Table 2, and the rover's traverse across the ridge with key waypoints illustrated is shown in Figure 4. *Curiosity*'s campaign at VRR was divided into three phases: approach, initial reconnaissance, and drilling. Rover activities during each phase are summarized below.

**Table 2:** Summary of Curiosity's Activities at VRR

	Earth date^a^	Sol	Activity
Approach imaging	6/14/17	1726	VRR approach imaging Stop 1
	6/15–6/21/17	1727–1733	Drive toward VRR approach imaging Stop 2
	6/22–6/24/17	1734–1736	VRR approach imaging Stop 2
	6/25–7/6/17	1737–1748	Drive toward VRR approach imaging Stop 3
	7/7–7/9/17	1749–1751	Eolian sediment investigation
	7/10–7/12/17	1751–1754	Continue drive toward VRR approach imaging Stop 3
	7/13–8/8/17	1755–1780	Solar conjunction
	8/9–8/10/17	1781–1782	Continue drive toward VRR approach imaging Stop 3
	8/11–8/12/17	1783–1784	VRR approach imaging Stop 3
	8/13–8/17/17	1786–1789	Drive toward VRR approach imaging Stop 4
	8/18–8/20/17	1790–1792	VRR approach imaging Stop 4
	8/21–8/24/17	1793–1796	Drive toward VRR approach imaging Stop 5
	8/25–8/27/17	1797–1798	VRR approach imaging Stop 5
	8/28–9/6/17	1799–1808	VRR approach
Initial walkabout; remote sensing and contact science focus	9/7/17	1809	Climb onto VRR; traverse Blunts Point/Pettegrove Point member boundary
	9/8–9/12/17	1810–1814	RS and CS measurements of Pettegrove Point
	9/13–9/17/17	1815–1819	Large fracture investigation
	9/17–11/2/17	1819–1864	RS and CS measurements of Pettegrove Point
	11/3–11/11/17	1865–1872	RS and CS measurements of Pettegrove Point/Jura member boundary
	11/12/17	1873	Traverse Pettegrove Point/Jura member boundary
	11/13–12/10/17	1874–1901	RS and CS measurements of Jura
	12/11/17–1/6/18	1902–1927	First investigation of gray Jura outcrop
	1/7–1/9/18	1928–1930	Drive toward second gray Jura outcrop patch; RS and CS measurements of Jura
	1/10–1/25/18	1931–1945	Investigation of large exposure of gray Jura
	1/26–1/29/18	1946–1949	Glen Torridon region toe dip and investigation of contact with ridge
	1/30–2/10/18	1950–1961	Investigation of additional gray Jura outcrop
	2/11–3/6/18	1962–1984	Unsuccessful rotary‐only drill attempt at Lake Orcadie
	3/7–3/24/18	1985–2002	Drives toward strong CRISM hematite signature; RS and CS measurements of Jura
	3/25/18	2003	Drive over Jura/Pettegrove Point member boundary
	3/26–3/31/18	2004–2009	RS and CS in area with strongest CRISM hematite spectral signature
	4/31–4/2/18	2009–2011	Investigation of Pettegrove Point/Jura member boundary
	4/3–4/4/18	2012–2013	Drive toward Bressay deposit
	4/5–4/14/18	2014–2022	Investigation of Bressay deposit
	4/15–4/24/18	2023–2032	Drives toward Blunts Point member; RS and CS measurements of Pettegrove Point
	4/25–4/27/18	2033–2035	Remote sensing of Taconite crater
	4/28/18	2036	Drives toward location of imaging of Red Cliff
	4/29–4/30/18	2037–2038	Red Cliff imaging
	5/1–5/6/18	2039–2044	Drives toward Blunts Point member; RS and CS measurements of Pettegrove Point
Duluth drill	5/7/18	2045	Drive over Pettegrove Point/Blunts Point member contact
	5/8–5/14/18	2046–2052	Search for Blunts Point drill target
	5/16–6/7/18	2053–2075	Duluth drill campaign begins
	6/8–6/17/18	2076–2085	MY34 Global Dust storm reaches *Curiosity*; Duluth drill campaign continues
	6/18–6/19/18	2086–2087	Conclusion of Duluth drill campaign
	6/20–6/26/18	2088–2093	Drive toward Pettegrove Point drill location
Pettegrove Point drill	6/27/18	2094	Drive over Blunts Point/Pettegrove Point member boundary
	6/28–7/11/18	2095–2108	Drive toward Pettegrove Point drill location; RS and CS measurements of Pettegrove Point outcrop
	7/12–7/17/18	2109–2114	Unsuccessful Pettegrove Point drill attempt at Voyageurs
	7/18–7/23/18	2115–2120	Drive toward Pettegrove Point drill Location 2; RS and CS measurements of Pettegrove Point outcrop
	7/24–7/28/18	2121–2125	Unsuccessful Pettegrove Point drill attempt at Ailsa Craig
	7/30–8/1/18	2126–2128	Drive toward Pettegrove Point drill Location 3; RS and CS measurements of Pettegrove Point outcrop
	8/2–8/4/18	2129–2131	Investigation of Pettegrove Point gray outcrop
	8/5–8/28/18	2132–2155	Successful Pettegrove Point drill at Stoer
Jura drill	8/29/18	2156	Drive toward gray Jura drill location
	8/30/18	2157	Drive over Pettegrove Point/Jura member contact
	8/31–9/9/18	2158–2166	Drive toward gray Jura drill location; RS and CS of Jura
	9/10–9/14/18	2167–2171	Unsuccessful gray Jura drill attempt at Inverness
	9/15–10/17/18	2172–2203	Memory anomaly precludes science operations
	10/18–10/27/18	2204–2213	Limited science operations begin
	10/28–10/29/18	2214–2215	Drive toward gray Jura drill Location 2
	10/30–10/31/18	2216–2217	Full science operations resume
	11/1–11/5/18	2218–2222	Drive toward gray Jura drill Location 2; RS and CS of Jura
	11/6–12/3/18	2223–2249	Successful gray Jura drill at Highfield
	12/4–12/11/18	2250–2257	Drive toward red Jura drill location; RS and CS of Jura
	12/12/18–1/20/19	2258–2296	Successful red Jura drill at Rock Hall
	1/21–1/25/19	2297–2301	Drives toward Glen Torridon; RS and CS of Jura
	1/26/19	2302	Depart VRR; enter Glen Torridon

^a^ Read Earth date 6/14/17 as 14 June 2017, etc.

Prior to landing, the science team divided 140 areas along *Curiosity*'s planned traverse in Gale crater into 1.5 km × 1.5 km (0.025°) quadrangles (Grotzinger, 2014). During the VRR campaign, *Curiosity* visited four of these quadrangles that were informally named “Bar Harbor,” “Kuruman,” “Torridon,” and “Biwabik.” Informal names for specific targets *Curiosity* observed were derived from rock formation names and local geographic names associated with the respective quadrangle where the target was observed.

### 4.1 Phase 1: VRR Approach Imaging (Sols 1726–1808)

**Figure 5:**
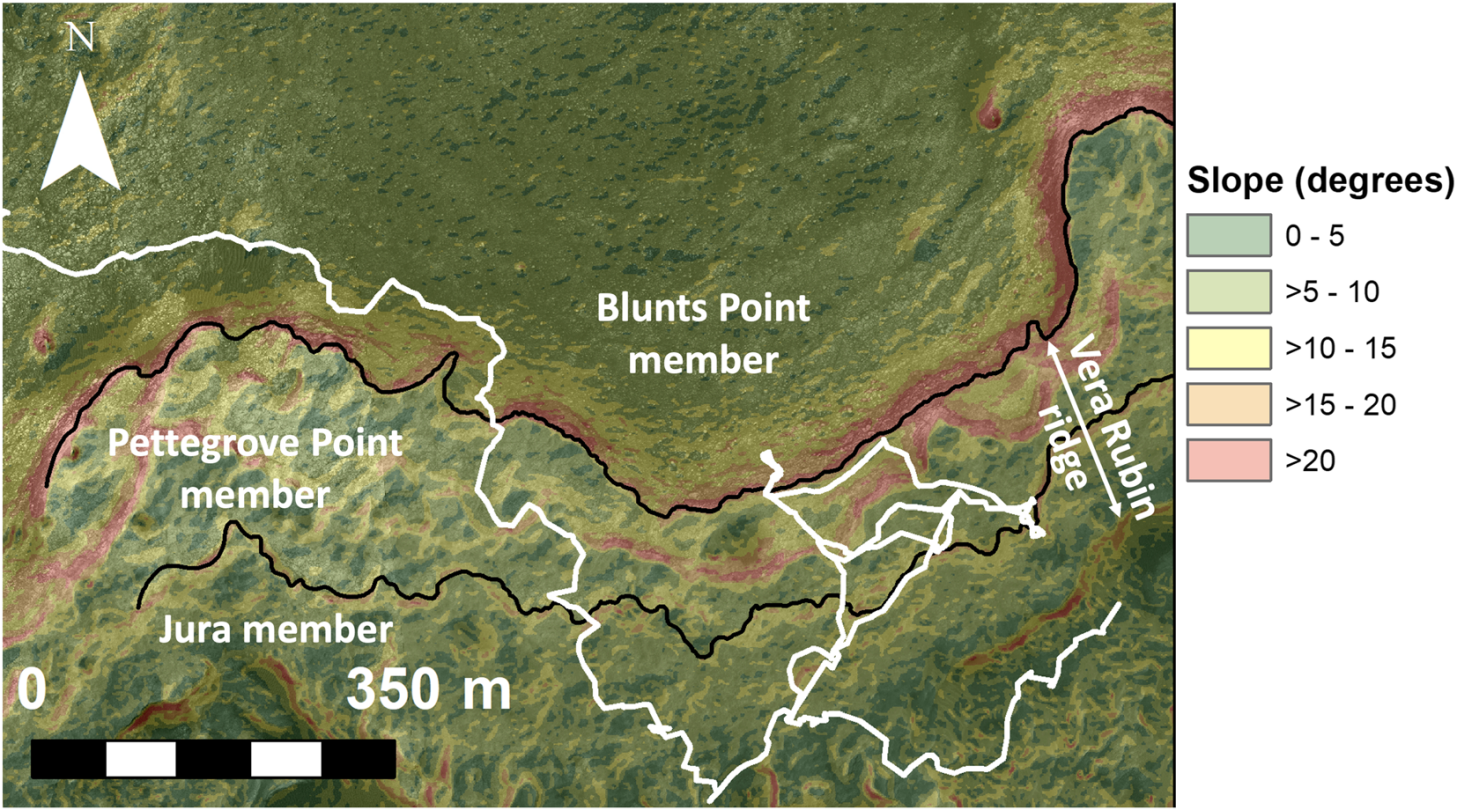
Slope map generated from HiRISE DEMs showing steep slopes bounding VRR. Black lines indicate stratigraphic member boundaries defined in Edgar et al. (2020).

**Figure 6:**
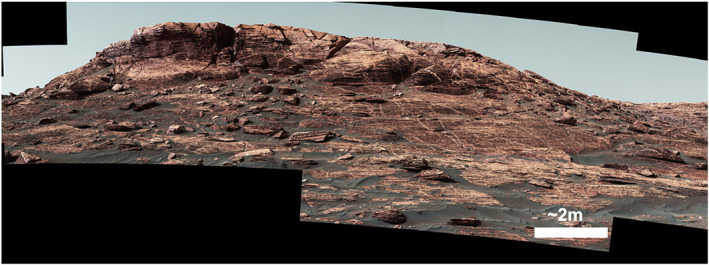
Example approach image Mastcam mosaic from Sol 1785 (sequence mcam09211).

*Curiosity* drove eastward along the base of VRR for ~430 m to access a location where slopes were shallow enough (<20–25°) for the vehicle to ascend (Figures 2 and 5). During this time, *Curiosity* acquired five large Mastcam M100 color mosaics that were pointed roughly south toward the base of the ridge (Table 3). The purpose of these mosaics was to document the transition between strata composing VRR and the underlying Blunts Point member and to image any sedimentary structures exposed on vertical faces in the lower portion of the ridge (Figure 6). *Curiosity* acquired the mosaics when the vehicle was between ~120 and ~35 m away from the ridge, which corresponded to M100 mosaic resolutions of ~90 to ~20 cm/pixel of the north side of the ridge, respectively. The rover also collected 11 RMI mosaics that provided higher resolution images of select sections of the ridge (Table 3).

**Table 3:** VRR Approach Mosaics

Sol	Instrument	Seq identifier	# frames
1726	Mastcam	mcam09012	18 × 1
1734	Mastcam	mcam09060	23 × 1
1785	Mastcam	mcam09211	35 × 2
1790	Mastcam	mcam09245	12 × 1
1797	Mastcam	mcam09282	44 × 1
1727	ChemCam RMI	ccam01727 VRR_Approach_Stop1	5 × 1
1734	ChemCam RMI	ccam02734 LD_Northern_Neck	10 × 2
1741	ChemCam RMI	ccam15121 LD_VRR_sol1741a	5 × 2
1741	ChemCam RMI	ccam15122 LD_VRR_sol1741b	5 × 1
1745	ChemCam RMI	ccam03744 VRR_LD_1745a	5 × 2
1745	ChemCam RMI	ccam03744 VRR_LD_1745b	5 × 2
1752	ChemCam RMI	ccam02752 LD_VRR_sol1752a	8 × 1
1783	ChemCam RMI	ccam01783 LD_VRR_sol1783a	5 × 1
1790	ChemCam RMI	ccam03790 LD_VRR_sol1790	10 × 1
1794	ChemCam RMI	ccam03794 LD_VRR_sol1794	5 × 1
1795	ChemCam RMI	ccam02795 LD_VRR_sol1795	10 × 2

### 4.2 Phase 2: Initial Reconnaissance With Remote Sensing and Contact Science (Sols 1809–2044)

As the rover's traverse steepened in the strata below the VRR, *Curiosity*'s strategic guidance had been to collect contact science measurements at least once every 5 m of elevation gain. These measurements included MAHLI textural observations and Alpha‐Particle X‐Ray Spectrometer (APXS) chemical observations on bedrock targets that were brushed beforehand using the dust removal tool (DRT) when images indicated significant dust coatings. The cadence of contact science sampling increased on Sol 1808 when *Curiosity* encountered a break in slope that marked the base of the ridge. The break in slope is accompanied by a lithological change where rocks are more competent and no longer dominated by the low‐angle Ca‐sulfate veins that are characteristic of the Blunts Point member. The decision to increase contact science data collection was motivated by the desire to capture chemical and textural changes that might be unique to the base of the VRR. *Curiosity* continued to sample VRR bedrock at <5 m elevation changes with contact science instruments as it traversed almost 1.5 km across the ridge during the next 200 sols. The rover also collected hundreds of remote sensing chemical and spectral measurements, predominantly of VRR bedrock targets, with ChemCam and Mastcam.

#### 4.2.1 Fracture Investigation (Sol 1814–1821)

The lower portion of VRR is crosscut by meter‐scale fractures that are visible in HiRISE data. Multispectral landscape images from several meters away showed that the fractures appeared to be associated with deeper ferric absorption bands at 535 and 867 nm. To assess whether the spectral differences were due to real compositional changes or an artifact caused by variable dust cover, *Curiosity* investigated material on the edge of the fracture on Sol 1815 and compared it with material from an area far from a fracture collected on Sol 1820. *Curiosity*'s observations included comparing the chemistry, spectral properties, and fine‐scale textures of brushed areas from both locations.

#### 4.2.2 Discovery and Investigation of “Gray Patches” (Sol 1902–1945)

**Figure 7:**
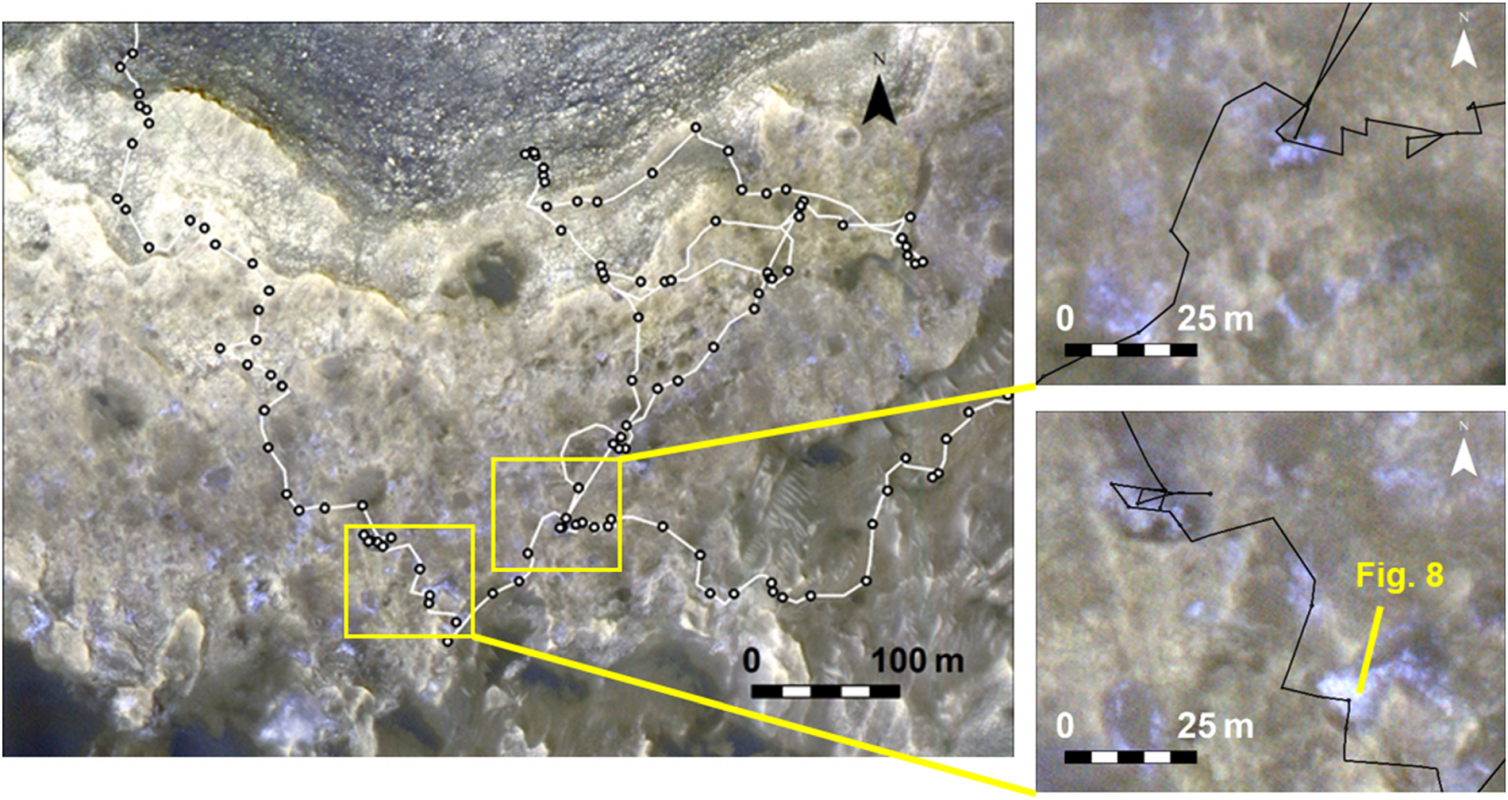
Stretched HiRISE mosaic from Fraeman et al. (2016) with color space adjusted to emphasize color variations along VRR. Inset boxes showing detail of “gray patches.”

Small areas on VRR appear blue‐gray relative to the surrounding terrain in stretched HiRISE false‐color images (Figure 7). These areas are primarily concentrated on the uppermost portion of the ridge, are ~1–10 m across, and are too small to resolve in CRISM data. Previous experience has shown that the underlying causes of color variations in HiRISE images are nonunique and could result from a variety of factors including changes in composition or texture or simply differences in amount of dust cover (Stack et al., 2016), so the rover was sent to investigate.

**Figure 8:**
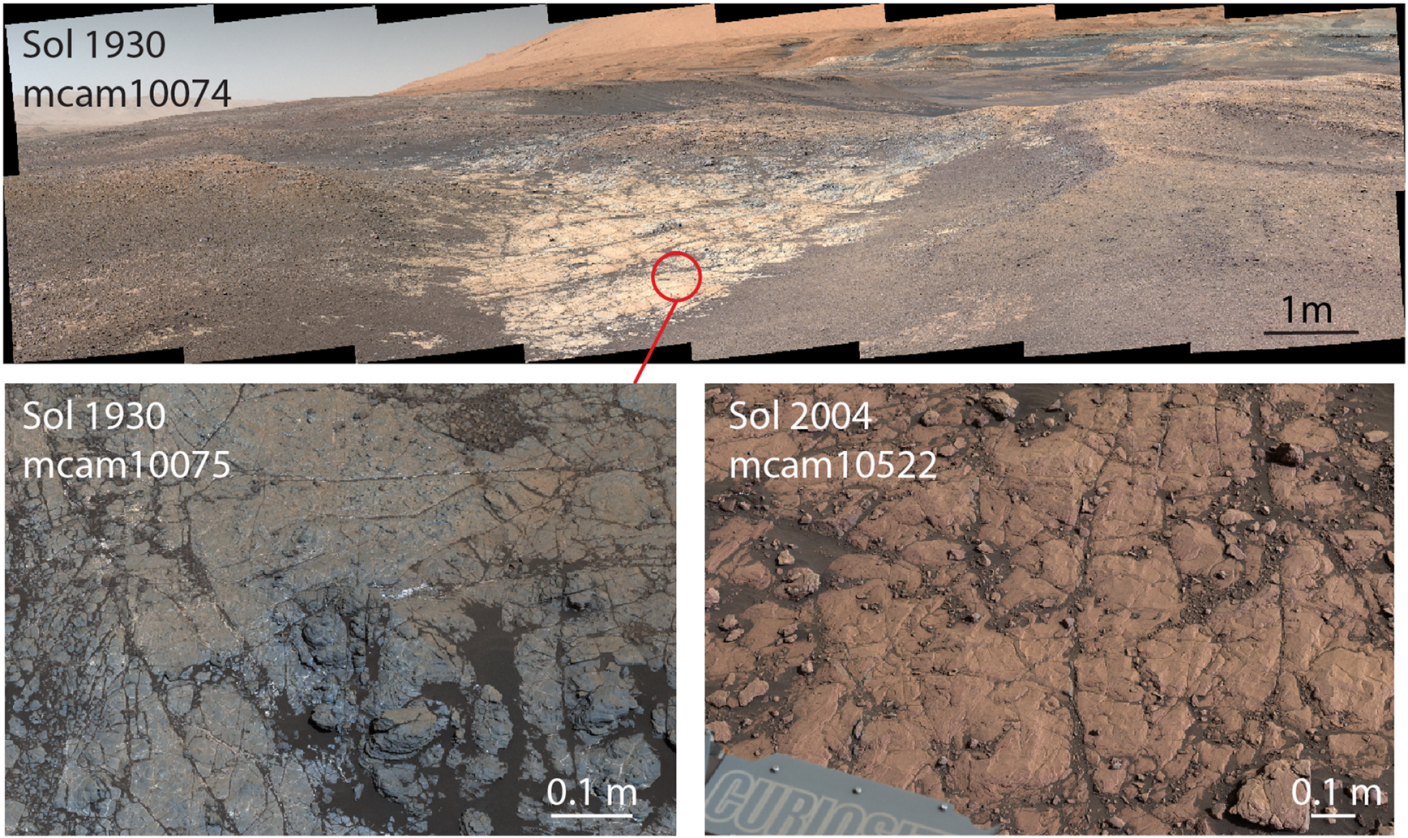
(top) Mastcam color standoff image of a recessive gray patch studied from Sol 1931–1945. The gray rocks appear tan from a distance because they are covered in light dust. They are surrounded by red pebbles of broken‐up red bedrock. (bottom left) Mastcam workspace image showing detail of gray rocks and (bottom right) comparison with red rocks lower on the ridge. Images are white balanced.

**Figure 9:**
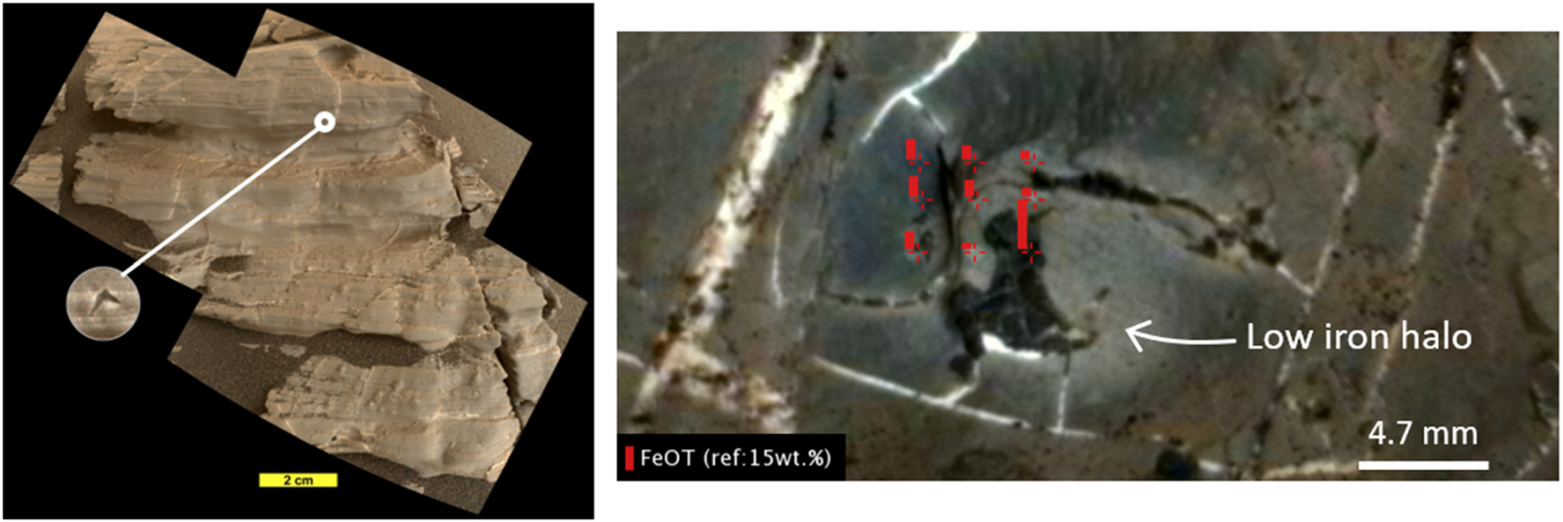
Examples of diagenetic features associated with gray areas. (left) Sol 1925 MAHLI 10 cm standoff mosaic of target “Jura” located in a transitional zone from red to gray bedrock. Example of swallow tail shown in circle (1926MH000369000070334(5,7,9)R00) inset circle is ~35 mm diameter. (right) RMI image of target “Rhynie” colorized with Mastcam that demonstrates an example of a nearly pure iron diagenetic feature (like iron oxide) within a gray patch surrounded by depleted iron bedrock. Length of red bars represents iron content measured by ChemCam reported in L'Haridon et al. (2020)*.*

*Curiosity* reached the first of these patches on Sol 1902 and discovered that the region is distinctly different in both texture and color compared with surrounding terrain (Figure 8) (Horgan, 2020). It is composed of competent gray bedrock, which contrast to the red pebbles that characterized the rest of the ridge. The gray bedrock often contains filled millimeter to centimeter‐sized dark, diagenetic nodules that are frequently associated with Ca‐sulfate filled veins that are ubiquitous throughout lower Mount Sharp (L'Haridon et al., 2018, 2020; Nachon et al., 2017). MAHLI images additionally revealed the first instances of millimeter‐sized crystal molds, some of which are filled and some of which are empty casts, near the red‐gray color transition (Figure 9).

We developed two hypotheses to explain the color and textural changes for bedrock in these areas. The first was that these color changes marked a sedimentary facies change and were related to differences in primary depositional environments. The second was that they were the result of variable diagenesis. In this scenario, the color changes could be due to diagenetically driven changes in composition or enhanced recrystallization. To test these hypotheses, we adjusted *Curiosity*'s strategic route to visit several more “gray patches,” including a particularly large region (Figure 8) that was directly to the south of the first discovery. *Curiosity* collected extensive images with Mastcam and MAHLI that documented the sedimentary textures within these regions, including lamination thickness as a function of stratigraphic position, and these images were examined for evidence of a facies change. *Curiosity* also collected extensive Mastcam multispectral, APXS, and ChemCam data from the gray and surrounding red rocks to search for chemical changes that might provide clues to how the features formed. Both the red and the gray rocks were also identified as high priorities for future drill sites, because mineralogy can potentially demonstrate differences not manifest in the bulk chemistry. *Curiosity* examined several of the millimeter‐sized dark diagenetic features and crystals with MAHLI, APXS rasters, and ChemCam LIBS observations to characterize their textures and compositions.

#### 4.2.3 Investigation at Area With Deepest 860 nm Absorption From Orbit (Sol 2004–2009)

**Figure 10:**
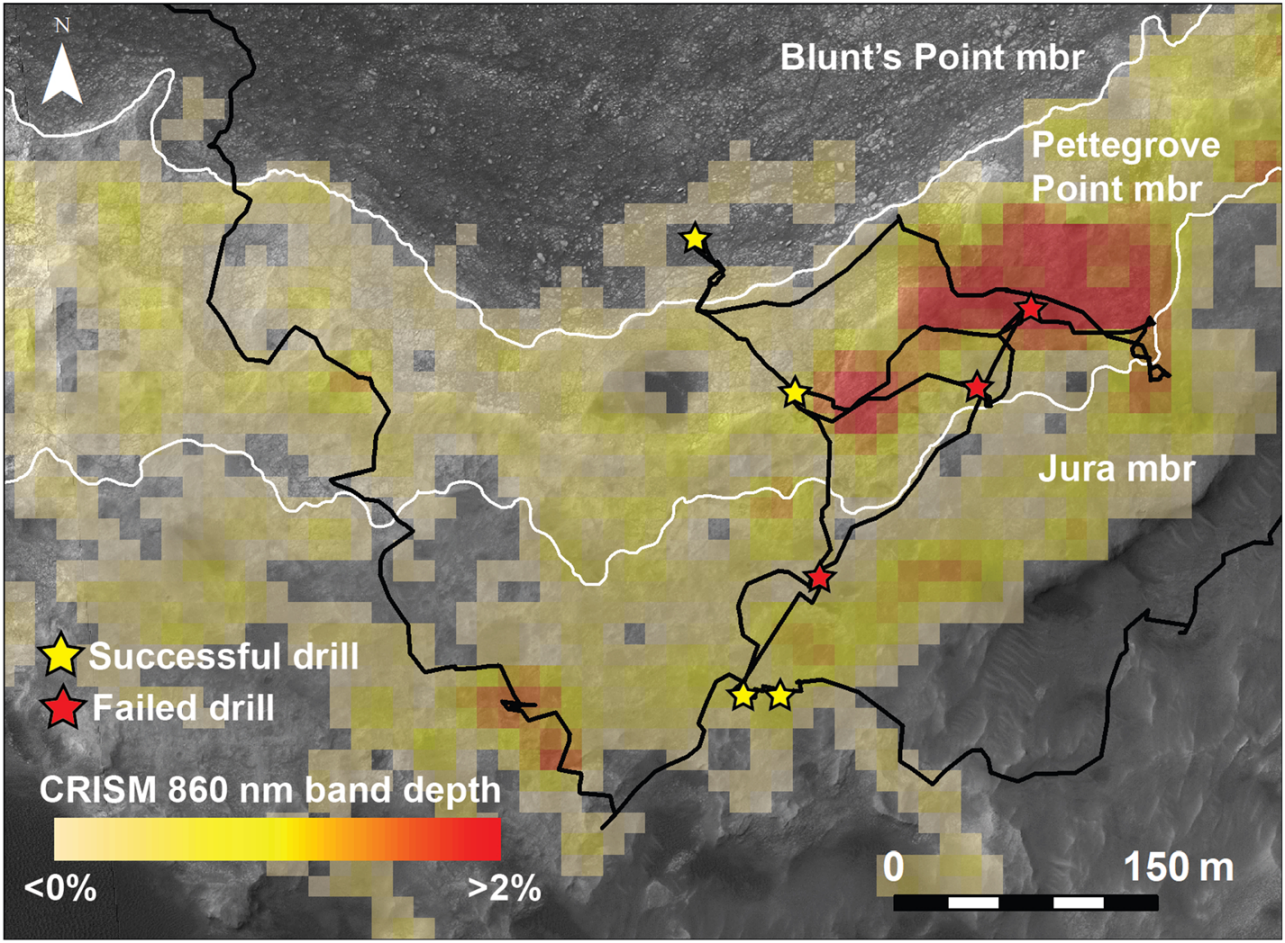
*Curiosity*'s traverse over VRR through Sol 2359 (black line) over color HiRISE mosaic, with CRISM 860 nm band depth map from ATO000021C92, stratigraphic members, and precussive drilling locations labeled.

*Curiosity* drove to an area on VRR that CRISM data show was associated with one of the deepest 535 and 860 nm hematite‐related absorptions to support the campaign goal of determining the source and geologic setting of the CRISM hematite signature (Figures 4 and 10). This excursion had the additional benefit of providing an east‐west transect along elevation contours for ~400 m that could highlight lateral variability within stratigraphically equivalent rocks. *Curiosity* reached the center of the “CRISM hematite hot spot” on Sol 2004 and began collecting extensive chemical, textural, and spectral data. *Curiosity* also took Mastcam multispectral images of the same terrain at multiple times of day that provided phase angle coverage from 0–130°. The purpose of these images was to investigate the photometric properties of the rocks in this area, including how the hematite‐relevant band depths (535 and 867 nm) varied as a function of lighting geometry (Johnson et al., 2019).

#### 4.2.4 Investigation at Bressay (Sol 2014–2022)

**Figure 11:**
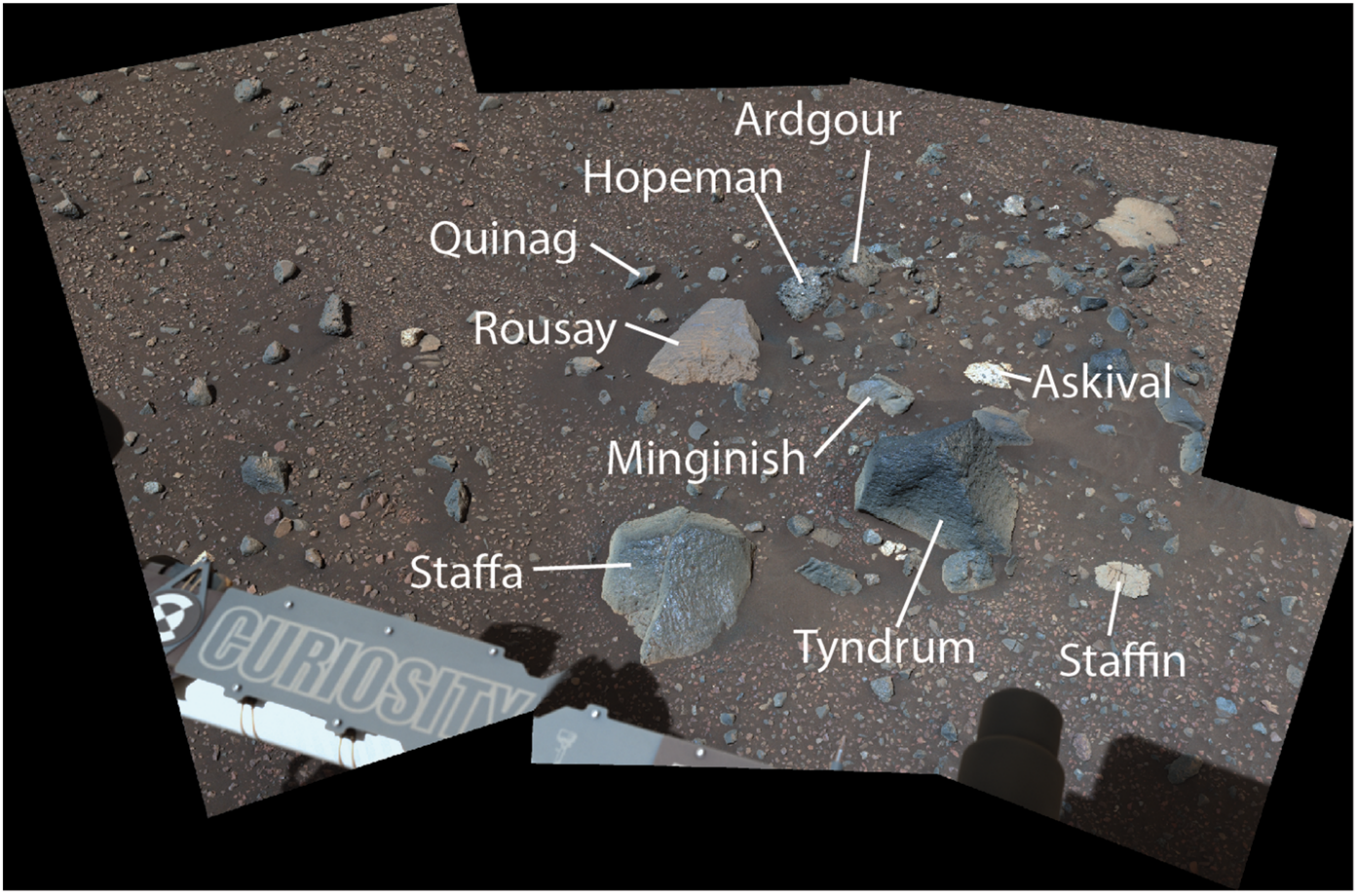
Mastcam 34 mosaic showing diversity of rocks in the Bressay deposit (Sol 2014, sequence mcam10630).

*Curiosity* investigated a collection of heterolithic float rocks designated as the “Bressay deposit” from Sols 2014–2022 (Figure 4, Point 12, and Figure 11). These rocks covered an area of ~3 m^2^ and had chemistries and textures that are distinct from VRR bedrock and other float rocks observed to date at Gale crater (Williams et al., 2020). These rocks must have been transported to this location on VRR, potentially from farther upslope via transport events associated with Gediz Vallis (Figure 1).

### 4.3 Phase 3: Drilling (Sol 2094–2302)

#### 4.3.1 Feed‐Extended Drilling and Sample Transfer

Two of *Curiosity*'s analytical instruments, CheMin and SAM, analyze powdered samples that were collected using the rover's rotary percussive drill. During nominal drilling activities, two stabilizers steady the drill against a fixed surface, while the drill's feed mechanism extends and retracts the drill bit relative to this surface. The same feed mechanism also transfers the powdered sample collected by the drill into the tool that sieves and portions the material (Anderson et al., 2012).

On Sol 1536 (December 2016), the drill feed mechanism began to exhibit intermittent failures. In response, rover engineers at the Jet Propulsion Laboratory commanded the drill to the fully extended position so that the bit would be clear of the stabilizers and remain usable in the event the feed mechanism failed entirely. They then developed new strategies for drilling and sample delivery that did not depend on the stabilizers or the sieving and portioning tool (NASA/JPL, 2018a). In this strategy, known as feed‐extended drilling (FED), the rover arm rather than the drill feed is used to extend and retract the drill bit into a surface, similar to how a human “freehand drills” with a power drill. Force sensors on *Curiosity*'s arm provide feedback to ensure the drill is not angled or at risk of getting stuck in the target. The powdered drill samples are then delivered to CheMin and SAM using feed‐extended sample transfer (FEST), which involves positioning the drill bit directly over the instrument inlets and rotating the bit in reverse. Extensive testing in *Curiosity*'s testbed on Earth showed this was safe in terms of portion amounts and particle size distribution.

FED was first tested on Mars on Sols 1977 and 1982 (February 2018) using rotary‐only capabilities in two nearby locations within a gray bedrock target on upper VRR named “Lake Orcadie” and “Lake Orcadie 2” (NASA/JPL, 2018b). While the tests demonstrated FED capabilities could be successfully implemented on Mars, the rotary‐only drill penetrated ~10 and ~2 mm in the targets, respectively, which was not deep enough for successful sample acquisition (~25–40 mm required).

With percussive drilling several weeks from being ready, the team took advantage of *Curiosity*'s position near the northern margin of VRR to descend the ridge with the goal of acquiring a sample from the Blunts Point member. The drill had not been available when *Curiosity* first traversed through that unit. The traverse path had the added benefit of acquiring observations along a second vertical traverse up the ridge that was laterally separated from the first. On Sol 2057 (20 May 2018) *Curiosity* successfully drilled the target “Duluth” using the new feed‐extended drilling using percussion (FED‐uP) technique, and samples from Duluth were transferred to CheMin a few sols later using FEST (NASA/JPL, 2018c, 2018d).

During drilling with percussion, percussive energy is provided by a voice‐coil mechanism that uses a magnetic field to oscillate a free mass, which acts as a hammer and transfers energy to the drill bit (Okon, 2010). Six unique voice‐coil levels on *Curiosity*'s drill can be used, with each level imparting greater energy into the surface. Voice‐Coil Level 1 is the lowest energy and 6 the highest. In FED‐uP drilling, the drill bit begins in rotary‐only mode and then autonomously steps up and down through the different voice‐coil levels based on the drill bit's measured rate of vertical progress (Abbey et al., 2019; Okon, 2010). The Duluth drilled sample, below the ridge, only required Voice‐Coil Level 2 for the drill to make sufficient rate of progress, qualitatively indicating that rock was not extremely hard (Table 4). In comparison, the drill required Voice‐Coil Level 5 to maintain a sufficient rate of progress for all of the successfully drilled samples on VRR. Due to a possible increased rate of actuator degradation at high percussion levels, the maximum allowable voice‐coil level was initially fixed at 5 for VRR drilling, although it was increased to 6 for Highfield and Rock Hall for the sake of saving mission time and accepting more risk.

**Table 4:** Summary of FED and FED‐uP Drills During the VRR Campaign

Sol	Target name	Stratigraphic Member	Elevation (m)	VCL reached (max VCL allowed)	Result
1977	Lake Orcadie	Jura (gray)	−4,147	Rotary‐only attempt	~10 mm penetration
1982	Lake Orcadie 2	Jura (gray)	−4,147	Rotary‐only attempt	~2 mm penetration
2057	Duluth (DU)	Blunts Point	−4,192	2 (5)	Success
2112	Voyageurs	Pettegrove Point	−4,164	5 (5)	~4 mm penetration
2122	Ailsa Craig	Pettegrove Point	−4,159	5 (5)	~5 mm penetration
2136	Stoer (ST)	Pettegrove Point	−4,170	5 (5)	Success
2170	Inverness	Jura (gray)	−4,150	5 (5)	~6 mm penetration
2224	Highfield (HF)	Jura (gray)	−4,147	5 (6)	Success
2261	Rock Hall (RH)	Jura (red)	−4,144	4 (6)	Success

*Note*. VCL = voice‐coil percussion level.

#### 4.3.2 VRR Drilling

All samples drilled on VRR were collected and analyzed using the FED‐uP and FEST techniques. Data from the reconnaissance phase of the campaign were used to prioritize what samples to collect while being mindful of finite rover and instrument resources. Drilling under FED/FEST is a particularly time‐intensive activity because *Curiosity* is precluded from using its arm for contact science activities or driving while the drill sample is held within the drill bit assembly. This means the rover has to stay parked at the drill location until sample delivery to CheMin and, if desired, SAM is complete.

The *Curiosity* team decided a minimum of three samples were needed to characterize the diversity of VRR rocks. VRR divides into two stratigraphic members defined by changes in lithology, shown in Figure 3 and discussed in more detail in results section 5.1. The lower member is named the Pettegrove Point member, and the upper is the Jura member. We decided one drill priority was a sample from the Pettegrove Point member, with a preference for that sample to be collected within the area associated with the CRISM pixels that had especially strong hematite spectral signatures. Two other high‐priority targets were red‐ and gray‐colored bedrock in the overlying Jura member. In order to assess the compositional changes and associated geologic processes responsible for the color differences, we desired to collect the red and gray samples that were as close to one another as practicable in order to minimize any effects of possible lateral and vertical variability in facies. For all drill targets, the team decided that to drill bedrock that had elemental compositions representative of the average compositions of these members would be preferable when possible.

**Figure 12:**
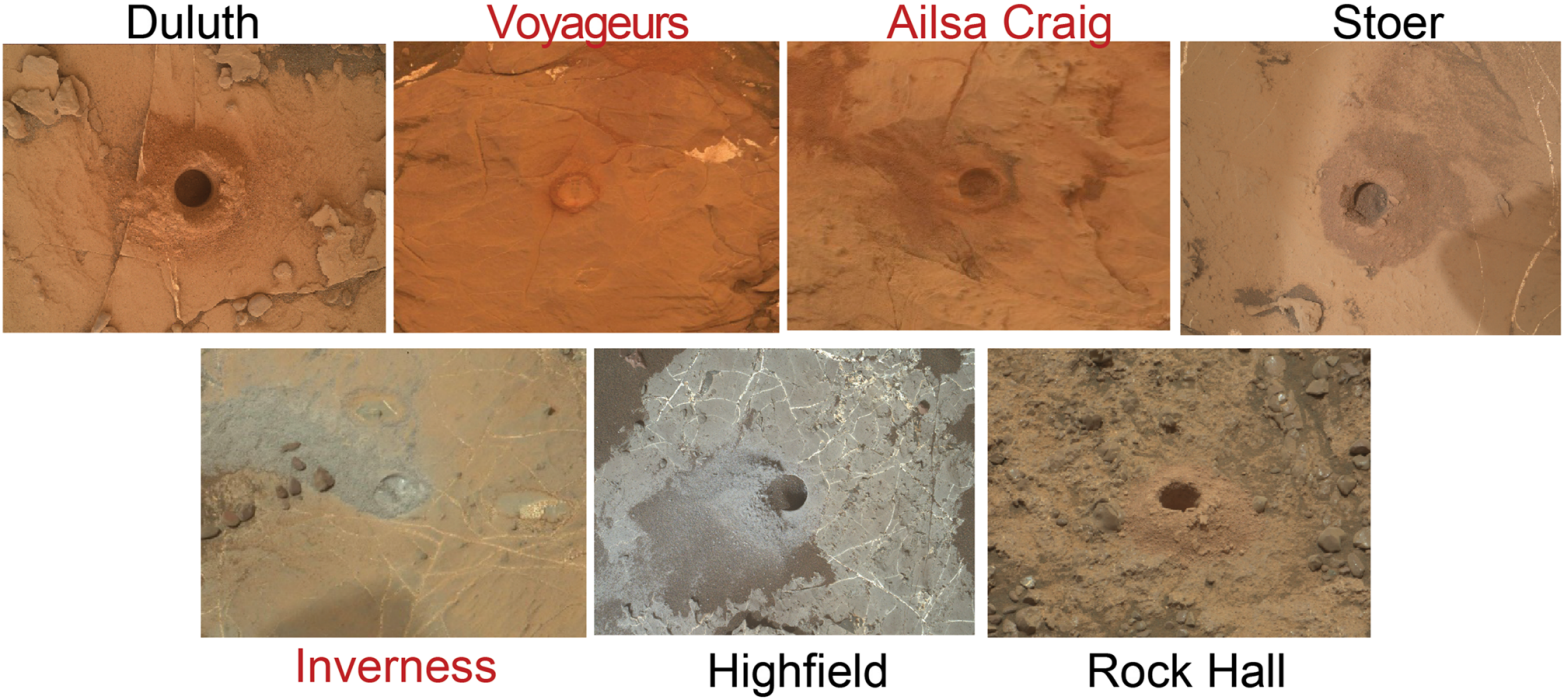
MAHLI and Mastcam images of drill attempts on and below Vera Rubin ridge. Diameter of drill hole is ~1.6 cm. Red target names indicate unsuccessful sample collection. Images IDs are the following: Duluth (DU): 2081MH0003970010801921C00, Inverness: 2114MR0113160000404710E01, Ailsa Craig: 2125MR0113930000404893E01, Stoer (ST): 2156MH0004240010802756C00, Inverness: 2171MR0116930010105700E01, Highfield (HF): 2247MH0004240010803292C00, Rock Hall (RH): 2262MR0120940080106571C00.

All attempted and successful drills during the VRR campaign are summarized in Table 4 and shown graphically in Figures 10 and 12. After Duluth, *Curiosity* attempted to drill the target “Voyageurs” on Sol 2112 in the area on the lower ridge (Pettegrove Point member) associated with the strongest CRISM hematite signature (Figures 4, 10, and 12). Despite rapidly increasing to percussion Level 5, the drill penetrated only ~4 mm before the rate of progress was deemed too slow and drilling operations autonomously ceased. *Curiosity* attempted to drill a second target ~60 m away named “Ailsa Craig.” This attempt similarly failed with only ~5 mm of progress even after reaching percussion Level 5. *Curiosity* then traveled ~110 m straight‐line distance to a third target, “Stoer.” Stoer still resides within the Pettegrove Point stratigraphic member, but it is not within a region associated with a strong CRISM hematite signature (Figures 4 and 10). We selected this target based on previous images of the area that showed the rocks here are more recessed compared to calcium sulfate fracture fills than typical Pettegrove Point rocks, which implied they might be slightly softer and more easily drilled. *Curiosity* successfully acquired a sample from Stoer on Sol 2136. The drill reached percussion Level 5 and maintained this level for ~200 s during drilling. Samples from Stoer were delivered to CheMin and to SAM several sols later.

*Curiosity* first attempted to drill a gray Jura target named “Inverness” on Sol 2170. This drill attempt also reached percussion Level 5 but only achieved ~6 mm of penetration before the rate of progress was deemed too slow and drilling ceased. The team decided to make a second attempt back in the Lake Orcadie region, ~80 m to the SW, because we were encouraged that one of the early, rotary‐only drill attempts there had made ~10 mm of progress. *Curiosity* successfully drilled a gray target, “Highfield,” near the previous Lake Orcadie attempted targets on Sol 2224. This Highfield drill required only 12 s of percussion Level 5 during the drill. Samples from Highfield were also delivered to both CheMin and SAM several sols later.

Searching for a red target near Highfield was challenging because most nearby red rocks were too small to drill. After a drive ended prematurely, *Curiosity* fortuitously discovered an outcrop ~35 m away from Highfield with rock slabs of sufficient size to remain stable during drilling. Although this outcrop has a slightly different texture and chemistry than typical red Jura rocks, we decided to still drill this target, named “Rock Hall,” because no obvious alternatives were in the vicinity. *Curiosity* successfully collected samples from Rock Hall on Sol 2261 and delivered them to CheMin and SAM on several sols later. The drill's voice‐coil only reached a maximum percussion level of 4 during drilling, suggesting this was one of the softest targets on the ridge.

## 5 Summary of VRR Key Findings

### 5.1 Primary Depositional Setting and Relationship With Mount Sharp

Rocks that make up VRR are predominantly composed of fine‐grained, thinly laminated parallel‐stratified bedrock that have approximately horizontal dips (Edgar et al., 2020, Stein et al., 2020). Sedimentary structures and textures observed throughout *Curiosity*'s traverse across the ridge are consistent with deposition in lacustrine and lacustrine margin settings, with a few isolated outcrops of low‐angle stratification that suggest possible subaqueous currents (Edgar et al., 2020). There is no evidence of an unconformity or depositional hiatus between the ridge and the underlying stratigraphic units in the approach mosaics that capture the vertical faces at the base of VRR (Table 3 and Figure 6), and rocks within VRR are therefore classified as members of the Murray formation (Edgar et al., 2020).

A stratigraphic column placing *Curiosity*'s results at VRR in the context of the rest of Mount Sharp is shown in Figure 3 and is discussed in detail by Edgar et al. (2020). In brief, rocks composing VRR are divided into the Pettegrove Point member and the overlying Jura member. The Pettegrove Point member is fine grained (mudstone to fine sandstone) and composed of parallel thin laminations. The Jura member is also a thinly laminated mudstone to fine sandstone. The Jura is distinguishable from the Pettegrove Point member by its darker color, tendency to erode consistently into centimeter‐sized clasts, and occurrence of local decimeter‐ to meter‐scale inclined strata that dip in multiple directions.

Although the Blunts Point, Pettegrove Point, and Jura members are defined from in situ data, these member boundaries are observable in orbital data as distinct changes in texture, color, and topography. As a result, the members can be traced laterally for kilometers beyond the rover's traverse. Although strata are generally horizontal within VRR, member boundaries mapped in situ by *Curiosity* and extrapolated to the orbital scale are not horizontal. For example, *Curiosity* crossed the same member boundaries (Blunts Point to Pettegrove Point and Pettegrove Point to Jura) at different elevations when the rover traversed them several hundred lateral meters apart (Edgar et al., 2020). The boundary between the Sutton Island and Blunts Point member also crosscuts elevation contours when traced in orbiter images, but *Curiosity* only crossed this boundary at once. The offset can be attributed to one of two explanations: VRR may have experienced differential compaction such that originally horizontal contacts are now slightly offset, or the contacts between these members record lateral variations in facies that would naturally vary with elevation as strata accumulate due to different inputs to the sedimentary basin(s) (Edgar et al., 2020).

At the time of publication (summer 2020), *Curiosity* is completing its investigation of the Glen Torridon (GT) region, which contains rocks characterized by a strong clay signature in orbital spectroscopic data, directly to the south of VRR (Figures 1 and 2) (Fox et al., 2019; Milliken et al., 2010). Results from preliminary geologic mapping of the GT area are important for placing VRR in stratigraphic context with the rest of the Mount Sharp group. *Curiosity* found evidence that similar facies appear at the top of VRR and within GT, and analyses of sedimentary structures show that strata within both areas formed in similar depositional environments. Combined with near‐horizontal dip estimates and elevation profiles across both units, these results show that the Jura member of VRR is stratigraphically equivalent to strata cropping out in the lower part of Glen Torridon (Stein et al., 2020). Mastcam and MAHLI images also show abundant veins, nodules, and crystal pseudomorphs, both empty and filled. These features are most dense in the gray patches and indicate multiple generations of fluid interaction at VRR (Bennett et al., 2018; L'Haridon et al., 2020).

### 5.2 Gray Patches: Due to Diagenesis, Not Facies Variation

**Figure 13:**
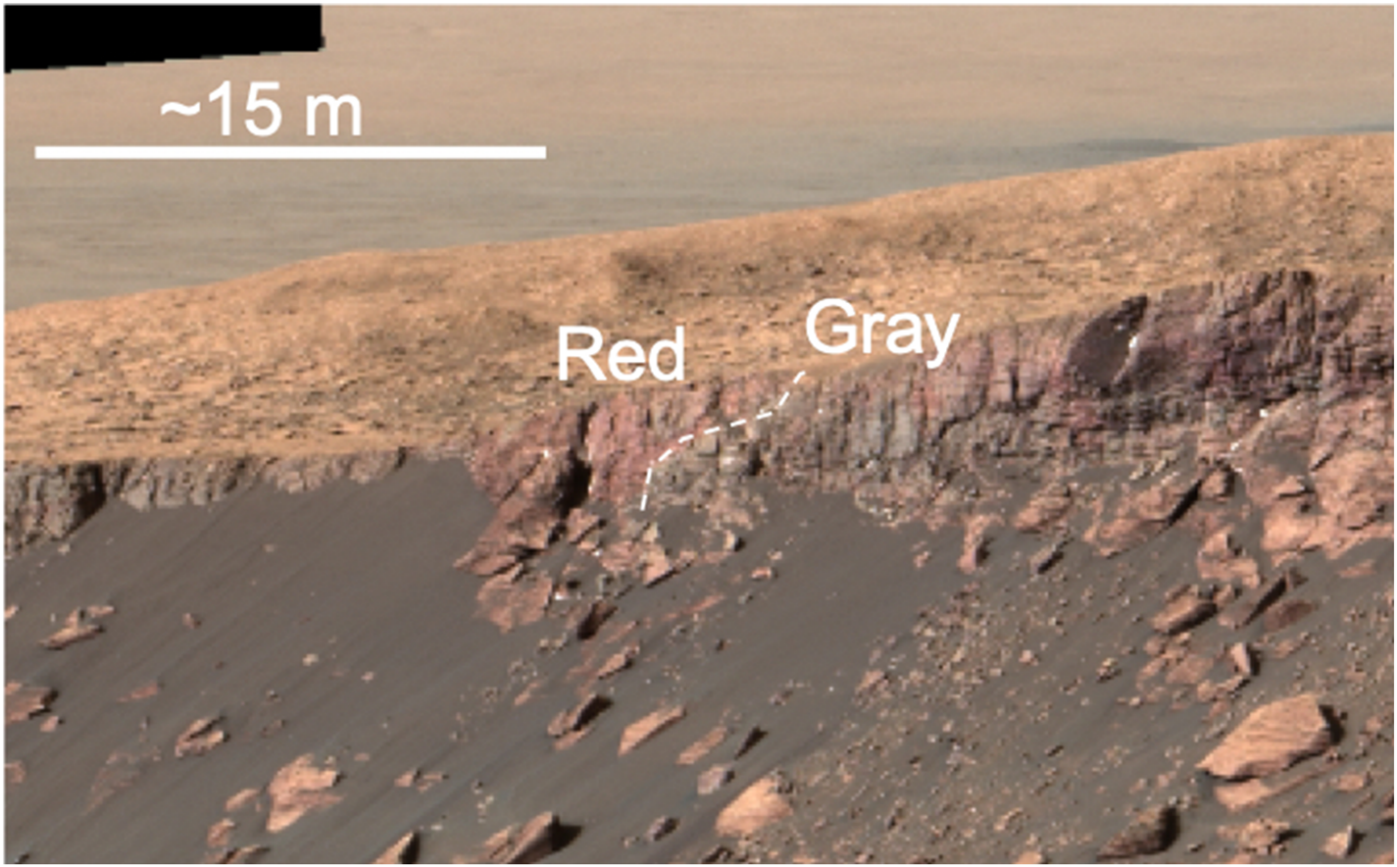
Portion of Mastcam R0 mosaic of Red Cliff area MR__578426886EDR_S0700240MCAM10769M showing red/gray color variations that cut primary bedding. Imaging has been stretched to highlight color variations.

The majority of gray VRR rocks *Curiosity* encountered occur within the Jura member, although the rover did observe an outcrop of gray rocks in the Pettegrove Point member on Sol 2128. Detailed observations of strata characterized by gray areas and surrounding rocks support the hypothesis that the color changes result from diagenetic processes rather than facies changes. Notably, sedimentary structures and textures do not change between the gray and red rocks within each member; sedimentary features in both are consistent with deposition by lacustrine processes (Edgar et al., 2020; Horgan, 2020). In many locations, the spectral transition from gray to red material is also gradational rather than discrete (Horgan, 2020). At least one example of a sharp color transition from red to gray is exposed on the vertical face of the Pettegrove Point member in an area informally named Red Cliff, and multispectral data show absorptions consistent with red and gray hematite (Horgan, 2020). Here the color variations clearly crosscut primary stratification (Figure 13).

### 5.3 Composition of VRR Compared With Underlying Murray Formation

#### 5.3.1 Elemental Chemistry From ChemCam and APXS

On the whole, the rocks within VRR have similar major element compositions to underlying Murray formation strata, excluding the Marias Pass locality (Table 5) (David et al., 2020; Frydenvang et al., 2020; Thompson et al., 2020). However, the ~50 m of vertical section *Curiosity* explored on VRR exhibits nearly as much chemical variability as the entire ~250 m of underlying Murray strata, again excluding the Marias Pass locality. Several papers in this special issue present detailed discussions of the chemical variability within VRR measured by APXS and ChemCam (Das et al., 2020; David et al., 2020; Frydenvang et al., 2020; L'Haridon et al., 2020; Thompson et al., 2020).

**Table 5:** Summary of Bulk Chemistry of VRR Compared to the Stratigraphically Underlying Murray Formation Measure by APXS and ChemCam

	APXS^a^				ChemCam^b^			
Oxide wt %	Baseline Murray^c^ (*n* = 162)	Std dev	Baseline VRR (*n* = 138)	Std dev	Baseline Murray^c^ (*n* = 2,915)	Std dev	Baseline VRR (*n* = 2,108)	Std dev
SiO_2_	48.39	3.23	47.86	2.52	53.4	2.6	54.4	2.1
TiO_2_	1.07	0.08	1.01	0.07	1	0.1	1.1	0.1
Al_2_O_3_	9.30	1.21	9.21	0.52	12.6	1.8	12.0	1.5
Cr_2_O_3_	0.33	0.04	0.31	0.02	—	—	—	—
FeO	18.88	2.79	18.10	2.54	19.2	1.5	19.7	1.3
MnO	0.23	0.10	0.20	0.09	—	—	—	—
MgO	5.59	1.17	5.49	0.71	5.8	2.1	4.7	0.9
CaO	4.31	1.29	4.65	1.03	2.2	1.0	2.0	0.6
Na_2_O	2.50	0.29	2.59	0.17	2.7	0.4	2.8	0.4
K_2_O	0.84	0.13	0.84	0.11	1.3	0.4	1.4	0.4
P_2_O_5_	1.04	0.27	0.86	0.15	—	—	—	—
SO_3_	6.08	2.33	6.08	1.81	—	—	—	—
Cl	1.07	0.52	1.39	0.49	—	—	—	—

*Note*. ChemCam data are not normalized to 100%, and minor differences between APXS and ChemCam reflect the differences in instrument field of view, sensitivity to dust, and actual targets sampled. Std dev = standard deviation.

^a^ Thompson et al. (2020).

^b^ Frydenvang et al. (2020).

^c^ Excludes targets with obvious diagenetic features, veins, and high Si targets from the Mariah's pass region.

One of the most significant findings at VRR was that, in spite of the ridge's strong spectral signature of hematite observed from orbit, neither APXS nor ChemCam observed increases in bulk FeO_T_ content in the bedrock that composes the ridge (David et al., 2020; Frydenvang et al., 2020; Thompson et al., 2020). However, near the Pettegrove Point and Jura member boundary, both instruments did measure MnO contents that were 2 times higher than the baseline values measured in typical Murray formation rock (Frydenvang et al., 2020; Thompson et al., 2020). While elevated, these were not the highest MnO values measured by either instrument in the Murray formation, which occur in the upper Sutton Island and lower Blunts Point members (Gasda et al., 2019; Frydenvang et al., 2020; Thompson et al., 2020). ChemCam also measured a clear drop in Li with increasing elevation on VRR (Frydenvang et al., 2020). Values of the chemical index of alteration (CIA, used to evaluate the extent of open‐system alteration; Nesbitt & Young, 1982) calculated from ChemCam data show a decrease toward the top of the ridge (Frydenvang et al., 2020).

The greatest chemical variability on VRR was observed within the “gray patches,” which APXS data show trend toward lower iron and higher aluminum and silica compared to average VRR rocks (Thompson et al., 2020). Using one quantification model, ChemCam data also show evidence for variable bulk FeO_T_ in bedrock in the Jura (David et al., 2020), although this is less clear with alternative calibrations (Frydenvang et al., 2020). ChemCam data also show small (approximately centimeter scale) areas within gray bedrock patches that have very low FeO_T_ which surround small (approximately millimeter scale) nodules that have nearly pure FeO_T_ (likely Fe_2_O_3_) compositions (Figure 9, David et al., 2020; L'Haridon et al., 2020). APXS also observed that a number of the gray areas are elevated in Se, with maximum values reaching up to 100 ppm (Thompson et al., 2020). For reference, average Se values throughout the Murray range from 0–20 ppm, although targets in the Pahrump Hills member have elevated values around 20–80 ppm.

#### 5.3.2 DAN Results

Several active DAN experiments were acquired within the Blunts Point, Pettegrove Point, and Jura members to assess the abundance of H and thermal neutron absorbing elements (e.g., Fe and Cl). These results are not summarized in any other papers within this special issue and so are discussed in detail here.

The DAN instrument footprint covers a ~1 m (full width half maxium) lateral area to a depth of ~45–75 cm (Mitrofanov et al., 2012). Bulk macroscopic neutron absorption cross section (*ξ*_abs_) and H content, reported as water‐equivalent hydrogen, for the materials within this field of view were derived according to the methods described in Gabriel et al. (2018). The *ξ*_abs_ parameter is positively correlated with the abundance of neutron absorbing elements, which are predominantly Fe and Cl on Mars (Hardgrove et al., 2011). Other species, such as Ni, Ti, Mn, and/or B may be important depending on their overall abundance and variability (Hardgrove et al., 2011). Multiple active neutron experiments were performed with the rover in a static configuration at 16 unique locations, and the time‐resolved spectra were coadded to improve signal‐to‐noise and counteract the degradation of the high‐energy neutron output over time from the DAN Pulse Neutron Generator (Sanin et al., 2015).

**Figure 14:**
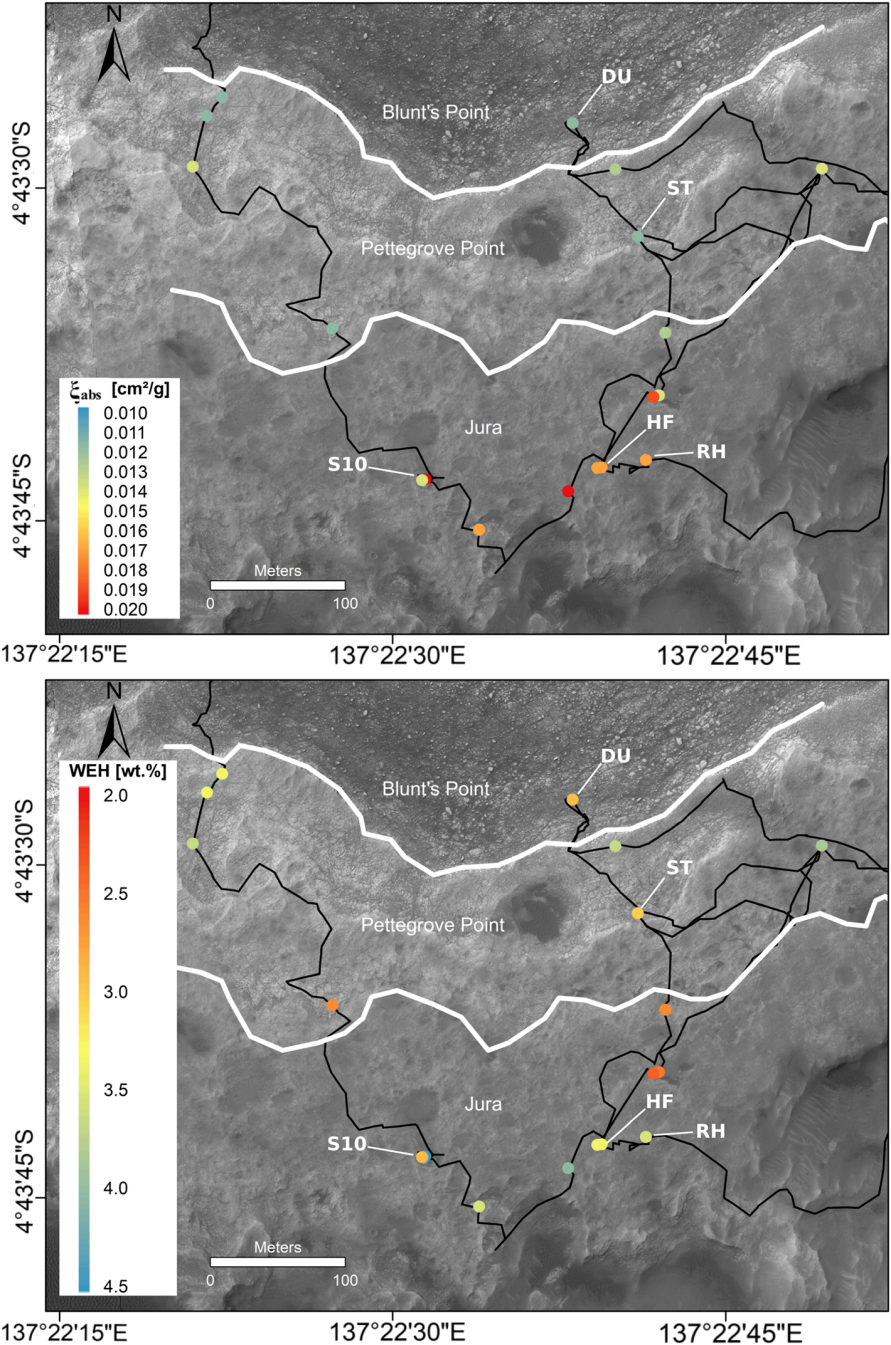
Top‐down maps at Vera Rubin ridge of (top) thermal neutron absorption cross section (*ξ*_abs_) and (bottom) water‐equivalent hydrogen (WEH) from analysis of active (time‐resolved) DAN experiments using the methods of Gabriel et al. (2018). Refer to Figure 15 for uncertainties. The black line represents the rover traverse between Sols ~1800 and ~2306. White lines represent the transition between members.

**Figure 15:**
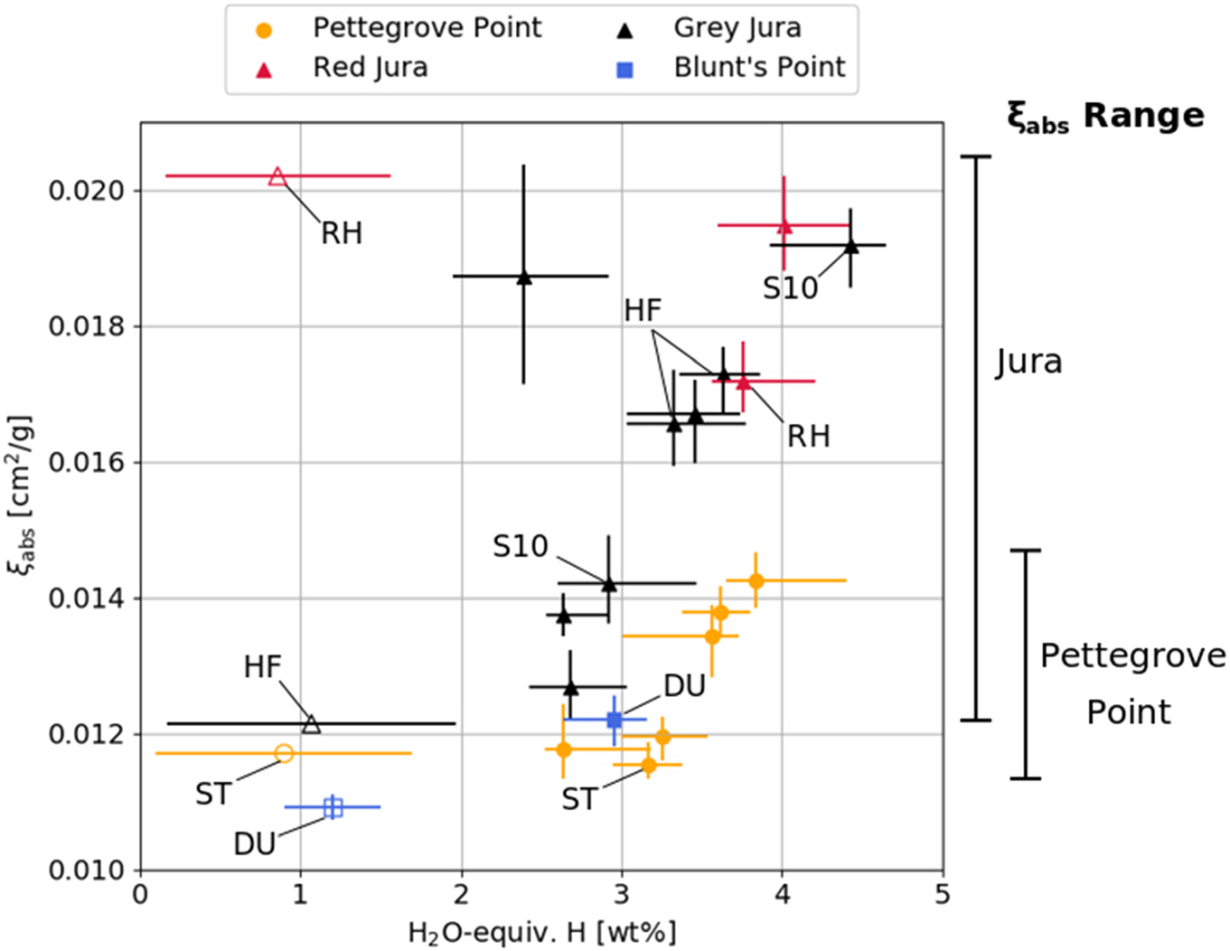
(filled symbols) Measured water‐equivalent hydrogen and thermal neutron absorption cross section (*ξ*_abs_) at drill sites from DAN data compared to (unfilled symbols) measurements from APXS of drill tailings (*ξ*_abs_) and SAM EGA of drilled samples (H_2_O). DAN points represent the median likelihood value, and error bars represent the 18% and 82% percentiles. Uncertainties for SAM/APXS data are at the 1 standard deviation level, and *ξ*_abs_ uncertainties on oxide abundances were only reported for the Duluth target in Thompson et al. (2020). The dynamic range of DAN‐derived *ξ*_abs_ in the Jura and Pettegrove Point members are shown on the right‐hand side. Labels: RH (Rock Hall drill target), HF (Highfield drill target), ST (Stoer drill target), DU (Duluth drill target), S10 (Site 10). We note that the active DAN experiments were not performed directly over the drill sites but were within 2–3 m.

We find the Jura member at the top of the VRR generally shows larger and more variable values of *ξ*_abs_ values than the lower Pettegrove Point member (Figure 14). For example, two active DAN experiments from within the Jura member ~3 m apart (at a gray patch informally called “Site 10”) show a difference in *ξ*_abs_ (see “S10” labels in Figure 15). Additionally, active DAN measurements near the Rock Hall (red Jura) and Highfield (gray Jura) sites show distinctly different values of *ξ*_abs_, indicating meter‐scale neutron absorbing element variability in that unit.

In contrast, no discernible trends were observed in the water content of VRR (Figure 14, bottom). Similar to other areas along the traverse, active DAN measurements consistently produced greater values of H than those derived from SAM experiments (McAdam et al., 2020) (Figure 15) potentially due in part to loss of hydrogen during to sample handling (Rapin et al., 2017). Dehydration has been observed in the CheMin instrument based on multiple observations of the same samples over a period of days (Vaniman et al., 2018). Some of the difference is possibly due to the scale of the DAN observation (~1 m lateral, ~45–75 cm depth) compared with the drilled samples (approximately centimeter scale, ~5.5 cm depth).

Attributing all the variability in *ξ*_abs_ (Figures 14, top, and 15) to changes in iron alone would require an absolute variability of ~25 wt % iron (throughout the entire DAN sensing volume) in the Jura; however, APXS and ChemCam analyses show that FeO_T_ contents vary on the order of ~5–15 wt % (David et al., 2020; Frydenvang et al., 2020; Thompson et al., 2020). Attributing the variability in *ξ*_abs_ to other minor neutron absorbers (Ni, Ti, and Mn) would require variations of ~10 wt % for Ni and Ti and ~5 wt % for Mn, which is well outside the range observed on the ridge by APXS and ChemCam analyses (Frydenvang et al., 2020; Thompson et al., 2020). Furthermore, Mn abundances trend toward lower values with increasing elevation in the Jura member (Frydenvang et al., 2020), opposite the trend in *ξ*_abs_.

Assuming the variability in *ξ*_abs_ within the Jura is due to Cl or B alone, changes of just ~1.25 wt % Cl or 160 ppm B could produce the observations. B is not observable with APXS, ChemCam has identified B enrichments in the VRR; however, ChemCam can only detect B in Fe‐poor materials that are small‐scale, light‐toned, diagenetic features, and the abundance of B has not been quantified (Das et al., 2020). Thus, the effect of B on the bulk (meter scale) rock *ξ*_abs_ is unknown. B is, however, anticorrelated with Li in Ca‐sulfate veins in the VRR (Das et al., 2020) and Li shows strong trends toward lower values with elevation (Frydenvang et al., 2020); concomitant increases of B in the bedrock would be qualitatively consistent with the larger average *ξ*_abs_ values observed in the Jura.

APXS measurements show that Cl abundances varied throughout the VRR, from 0.4–2.7 wt %, (Thompson et al., 2020), consistent with the range of DAN‐derived *ξ*_abs_ values. Some Cl is likely hosted in a Cl‐bearing iron oxide‐hydroxide mineral, akaganeite, that was detected in the Stoer and Rock Hall drilled samples (Rampe et al., 2020). However, not all of the Cl measured by APXS is taken into account by akaganeite abundance as measured by CheMin, and Cl is likely also variably present in salts and within XRD amorphous materials in all three VRR drilled samples, although salts, if present, are below CheMin detection limits (McAdam et al., 2020; Rampe et al., 2020).

In summary, active DAN investigations at VRR are consistent with results from other payload instruments that show Cl and, to some extent Fe materials, are heterogeneously distributed within the Jura member. DAN data are also consistent with a heterogeneous distribution of neutron absorbing elements (i.e., Cl, B, and/or Fe) at the meter scale and are especially variable within gray Jura bedrock patches. Additional colocations of active DAN footprints with APXS and ChemCam measurements are necessary to further pinpoint the exact source of large‐scale variability in neutron absorbers across the ridge and thus characterize the relative mobility of Cl versus Fe species in diagenetic events.

#### 5.3.3 Drilled Sample Analyses

The three drilled samples from VRR were selected to represent the diversity of VRR rocks discussed in section 4.3.2. The Pettegrove Point member was sampled at the target Stoer, and the gray and red Jura members were sampled at the Highfield and Rock Hall targets, respectively. As mentioned in section 4.3.2, there was some uncertainty whether the Rock Hall drilled sample was representative of the bulk of the red Jura member. APXS analysis of Rock Hall of drill tailings were elevated in Ca, S, Cl, and Br compared to other Jura targets and also had more Fe and Ni (Thompson et al., 2020). ChemCam analysis also showed nearby rocks had higher SiO_2_ and CIA than rocks lower on the ridge (Frydenvang et al., 2020).

Rampe et al. (2020) and McAdam et al. (2020) describe in detail the CheMin‐ and SAM‐derived compositions, respectively, of VRR drilled samples. CheMin data show all three VRR samples contain feldspars, pyroxene, hematite, calcium sulfates, phyllosilicates, and X‐ray amorphous material. The Highfield sample from the gray Jura has a very similar crystalline mineralogy as the Stoer red Pettegrove Point sample. The red sample has the most hematite of any sample drilled to date (~15 wt % of the bulk), although the hematite abundance is not significantly greater than rocks in the underlying Murray formation. Hematite (~9 wt %) is present in the gray sample, which, when combined with the color and spectral properties of this material (Figure 12), is interpreted to imply gray rather than red hematite (>5 μm crystals) is present (Morris et al., 2020 *;* Rampe et al., 2020). Spectral properties of the gray patches and dark diagenetic features within are also consistent with gray hematite (Horgan, 2020). Hematite is the dominant iron oxide in Stoer and Highfield, and these samples also contain around 0.5 wt % magnetite. For Rock Hall, akaganeite is the dominant iron oxide with minor hematite and no detectable magnetite. Stoer and Rock Hall also have minor jarosite, ~1 and ~2 wt %, respectively.

SAM evolved gas analyses corroborate the CheMin Fe‐rich phyllosilicate detection, and also show all samples contained amorphous Mg sulfates (McAdam et al., 2020 *e*), which are not detected as crystalline phases by CheMin. Trace and/or amorphous reduced sulfur species, either iron sulfides or S‐bearing organic compounds, may also be present in the Highfield and Rock Hall samples, but at abundances far below the CheMin detection limit (Wong et al., 2020). SAM also showed all three samples contained trace chloride salts and that Rock Hall also revealed evidence for oxychlorine and nitrate salts. Oxychlorine compounds had not been observed for ~1,200 sols (McAdam et al., 2020).

Turner et al. (2020) use thermochemical modeling based on CheMin and APXS analyses to demonstrate that the clay‐hematite assemblage observed on and below VRR could be formed through alteration by dilute groundwater brines with high water/rock ratios that are higher than Yellowknife Bay (Bridges et al., 2015). In this model, later alteration phases including the sulfates and akaganeite were superimposed on the main clay‐hematite assemblage associated with VRR.

### 5.4 Spectral Variability and Links to CRISM Observation

CheMin XRD data coupled with Mastcam multispectral and ChemCam passive spectral observations show that red hematite is dispersed throughout much of the VRR bedrock and is thus the source of the ~530 and ~860 nm spectral absorptions observed by CRISM over this region (Fraeman et al., 2020; Horgan, 2020 *;* Rampe et al., 2020). Synergistic analysis of orbital and in situ spectral data sets demonstrates that VRR is associated with comparatively deep hematite‐related spectral absorptions in orbital data in part because less sand and dust obscure the ridge but also because, in several places, the VRR bedrock is associated with stronger spectral absorptions at ~530 and ~860 nm than observed anywhere else in the Murray formation (Fraeman et al., 2020). CheMin, ChemCam, and APXS data all support the hypothesis that these deeper hematite‐related spectral absorptions are primarily due to changes in grain size and/or a higher proportion of total hematite presenting as pigmenting hematite (Frydenvang et al., 2020; Jacob et al., 2020; Horgan, 2020; Rampe et al., 2020; Thompson et al., 2020). Notably, the spectral observations at VRR do not reflect significantly greater abundances of ferric minerals at VRR, which does not support the original interpretation of VRR as being a site of substantial iron enrichment. Maps of multiple spectral properties (band depths, slopes) across the ridge show that variations in these properties crosscut the primary sedimentary stratification, indicating that diagenetic alteration is likely responsible for the large‐scale spectral variability, including within regions on VRR that have remarkably deep hematite‐related spectral absorptions (Horgan, 2020).

*Curiosity*'s investigation of the fractured outcrop on the lower ridge from Sols 1814–1819 revealed no significant chemical or spectral differences between outcrop near the fractures versus fracture‐free outcrop once dust was removed. The apparent enhancement in hematite‐related absorptions observed in the Mastcam multispectral landscape images of the area occurred because the rough surfaces that bound the fractures are less dusty than the smooth surfaces between fractures (Fraeman et al., 2020).

### 5.5 Rock Hardness Within VRR

Two metrics qualitatively demonstrate that the rocks of the ridge are stronger than surrounding strata. First, the ridge itself stands topographically higher than surrounding rocks and is therefore more resistant to erosion than surrounding rocks. A distinct break in slope on both the north and south sides of the ridge results in it standing tens of meters above surrounding terrain (Figure 2). Second, *Curiosity*'s drilling activities failed at several locations on the ridge because the hardness of the rocks was too great for the drill to achieve a sufficient rate of downward progress. Two of the three successful drill holes on VRR required a maximum percussion voice‐coil level of 5, while the percussion levels on the third drill hole reached Level 4. In comparison, *Curiosity*'s drill holes in rocks immediately to the north (Duluth) and south of the ridge (Sol 2369's “Aberlady” and Sol 2384's “Kilmarie” in Glen Torridon) only required a maximum percussion level of 2. For reference, voice‐coil levels of 2, 4, and 5 correspond percussion mechanism impact energies of 0.20, 0.45, and 0.61 J, respectively (Peters et al., 2018). Methods to quantitatively estimate the compressive rock strengths using drill telemetry for nominal *Curiosity* drilling have been developed (Peters et al., 2018), but they are not applicable to FED‐uP drilling. However, the significantly different percussive levels required to drill VRR compared with surrounding units is a convincing qualitative indicator that VRR rocks are comparatively hard.

## 6 Synthesis: The Origin of VRR

### 6.1 Reason for Relative Erosion Resistance of VRR

The sedimentary rocks that compose VRR form a ridge because they are stronger and more resistant to erosion than the rocks in the surrounding terrain. The induration of sedimentary rocks is primarily affected by compaction and cementation, which in turn is linked to porosity and permeability (e.g., Burley & Worden, 2003). On average, the individual grains that compose the VRR rocks and the rocks beneath VRR are smaller than the maximum resolving power of MAHLI, which is ~17–45 μm (Bennett et al., 2018; Edgett et al., 2012). *Curiosity*'s instruments therefore cannot directly observe if grain size or shape differences are exclusive contributors to the difference in strength between VRR and adjacent rocks. In lieu of direct measurements, grain sizes can be estimated using the Gini index, a statistical parameter that describes the point‐to‐point variability of ChemCam LIBS points (Rivera‐Hernández et al., 2019). Calculated Gini index values for rocks in the Pettegrove Point and Jura members are similar to values from rocks in the recessive Blunts Point member rocks, suggesting only a slight coarsening upward from the Blunts Point member through the Jura (Bennett et al., 2018). Given this result, it seems likely that enhanced cementation played at least some part in VRR's relative resistance to erosion compared with underlying and adjacent strata. Increased cementation at VRR is also consistent with its higher thermal inertia compared with surrounding Murray formation in orbital data sets (~350–400 vs. ~200–250 J m^−2^ K^−1^ s^−1/2^) (Edwards et al., 2018).

What is the composition of the cement in VRR rocks? The association of the orbital hematite spectral signature with VRR was initially interpreted to suggest hematite as the cementing agent (Fraeman et al., 2013). However, CheMin data do not show any correlation between qualitative rock strength and crystalline hematite abundance (Jacob et al., 2020). Furthermore, there is no obvious correlation between qualitative rock strength and abundance of any crystalline phases measured by CheMin, elemental compositions measured by APXS or ChemCam, or estimated elemental compositions of the amorphous material in the CheMin data (Jacob et al., 2020). Mechanical studies show that very small amounts of cement can increase the strength of granular materials (Dvorkin et al., 1994; Wang et al., 2019; Yin & Dvorkin, 1994). Small changes in amount and composition of cement may therefore be undetectable by *Curiosity*'s payload instruments but still contribute to the increased strength of VRR rocks.

Below VRR, quantitative calculations of the compressive strength of Murray formation rocks drilled with nominal drilling techniques similarly showed no clear correlations with CheMin‐measured crystalline mineralogy or APXS/ChemCam elemental compositions (Peters et al., 2018). Based on the phases that were present, Peters et al. (2018) hypothesized either hematite, calcium sulfate, and/or phyllosilicates could be effective chemically derived cementing material in the Murray formation, and Smith et al., (2020) investigated the possibility of early stage diagenetic silica. These phases may also be cementing VRR.

### 6.2 *Curiosity*'s Findings Eliminate Several Orbital‐Based Hypotheses for VRR's Origin

Data collected during *Curiosity*'s campaign at VRR do not support several hypotheses previously proposed about the ridge's origin that were proposed from orbital observations (summarized in section 1). If the ridge formed at a redox interface where dissolved Fe (II) was transported by near‐neutral, anoxic waters that were later oxidized and caused precipitation of Fe (III) phases, we would expect to see either an increase in the total measured amount of iron at the ridge compared with surrounding regions or a substantial increase in the total wt % of ferric minerals. Neither is observed, so the redox interface hypothesis in not supported by VRR in situ data.

*Curiosity* data also definitively confirm that the ridge is not an area that experienced extensive, top‐down oxidative weathering that would have left a lag of iron oxides and other insoluble phases, similar to a laterite deposit. If this process had occurred, APXS and ChemCam would have measured increases in iron, aluminum, and titanium with increasing elevation on VRR (i.e., Nesbitt & Young, 1982). Similarly, the elemental enrichments and depletions caused by strong oxidative weathering would have been evident in increasingly higher calculated CIA values with increasing elevation on the ridge. Instead, APXS show CIA values that are constant across VRR, and ChemCam data even suggest that CIA values decrease toward the top of ridge, with values around 50–55 compared to the underlying Murray formation where values frequently reached 60 or more (Frydenvang et al., 2020; Mangold et al., 2019). This observation is directly opposite what would be observed in a scenario where open‐system weathering was concentrated at the top of the ridge due to subaerial exposure. CheMin also did not observe any greater abundances of minerals that form in highly weathered environments, such as aluminous clays and silica phases, which would have been expected in this scenario (Rampe et al., 2020).

The findings at VRR neither refute or support a model where ferric or mixed ferrous/ferric precursor, such as green rust or ferrihydrite, precipitated directly in the lake at Gale (Hurowitz et al., 2017; Tosca et al., 2018). However, this model by itself does not explain VRR, that is, VRR is not an isolated region where ferric phases precipitated directly in a lacustrine setting in response to changing redox conditions. In such a setting, we would expect Fe‐rich bands and hematite spectra that followed primary bedding. In contrast, variations in hematite spectral signatures crosscut stratal boundaries (Fraeman et al., 2020), and measured FeO_T_ contents do not vary in a systematic way between strata (Frydenvang et al., 2020; Thompson et al., 2020). If ferric phases did precipitate directly in a lake, they were likely recrystallized and/or supplemented by additional ferric phases that formed during later diagenesis.

### 6.3 Postdepositional Processes Shaped VRR

**Figure 16:**
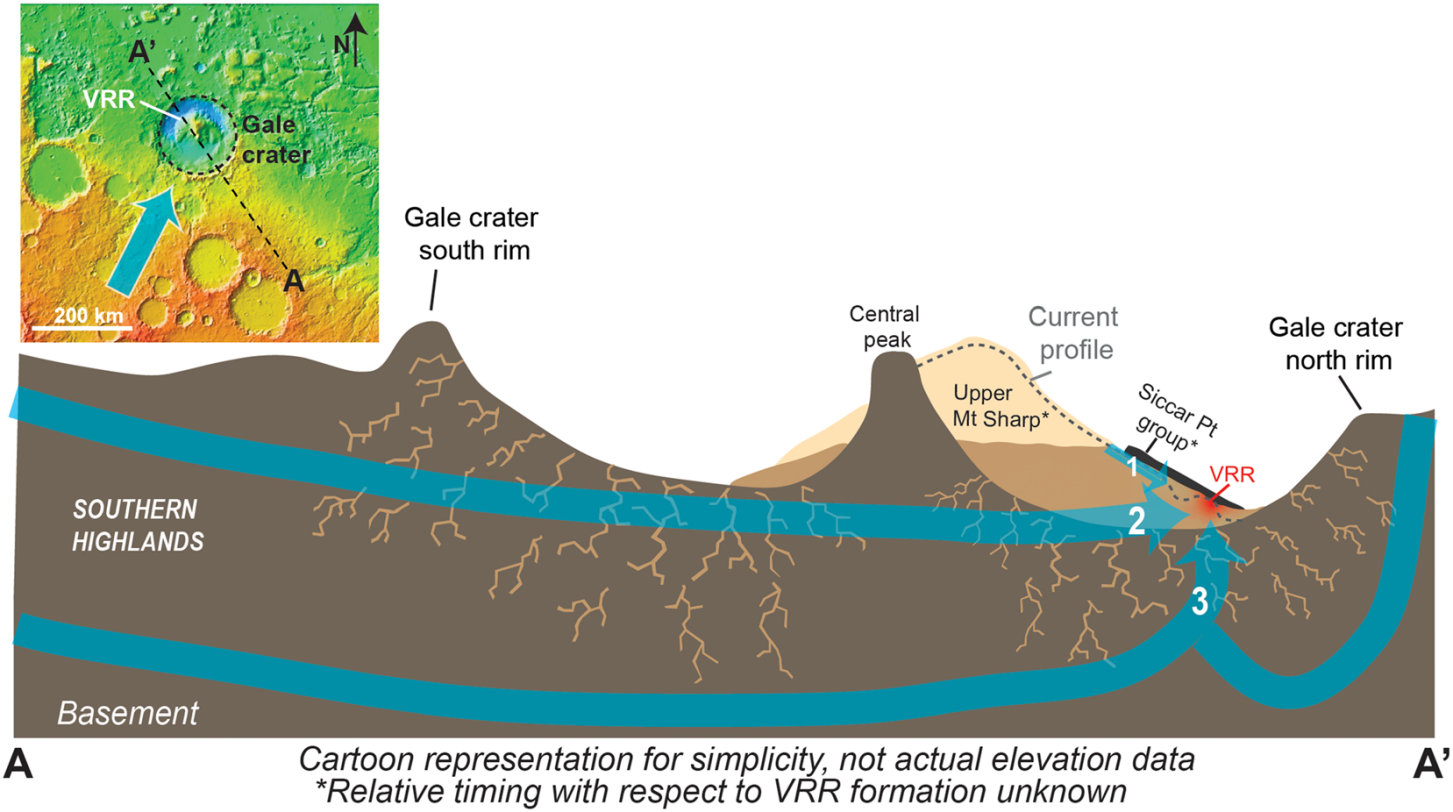
(upper left) Regional topographic map with representation showing regional flow (blue arrow in inset) that likely passed into and across Gale (Irwin et al., 2005; Palucis et al., 2016). (bottom) Idealized cross section of Gale crater (distorted scales) locating Vera Rubin ridge (VRR) at the base of Mount Sharp and the representing the possible major pathways of water that may have contributed to diagenesis of the sediments. At the time of diagenesis VRR was not fully exposed as it is today. Note that the cross section A‐A′ lies approximately perpendicular to hypothesized Paths (2) and (3), but a component would have been in the paths shown. Path (1) follows relatively shallow subsurface flows off Mount Sharp that may have been directed down Gediz Vallis toward the current location of VRR. Path (2) depicts a path of groundwater that drains from the southern highlands into Gale, crossing Mount Sharp. Path (3) shows that in the floor of Gale, the deeper pathways of the drainage from the south or local infiltrating waters can ascend to the surface.

We propose the VRR topography formed by wind erosion of a ~200 m wide, ~6.5 km long band of rocks along the base of Mount Sharp that had been preferentially hardened by diagenetic processes (Figure 16). Diagenesis in a mostly closed system caused enhanced crystallization and/or cementation that was associated with only minor compositional changes. However, this process changed the mineral grain size/crystallinity of some ferric phases (as evidenced in part by sharpened hematite diffraction peaks in CheMin data (Rampe et al., 2020) but below resolutions detectable using the Gini Index). This is also consistent with Mastcam spectral properties suggesting a gradual coarsening of hematite through VRR, from finest in Blunts Point and coarsening through Pettegrove Point, red Jura, and coarsest in the gray Jura (Horgan, 2020). This coarsening/recrystallization resulted in the deep ferric‐related spectral absorptions that are so distinguishable on the ridge from orbit. Formation of VRR by this model is also consistent with the finding that the ridge is geomorphic feature but not sedimentologically or stratigraphically distinct from the underlying and laterally equivalent Murray formation rocks.

In addition to hardening the rocks that compose the ridge itself, diagenesis at VRR also created abundant centimeter‐scale textural features (e.g., veins, nodules), meter‐scale gray patches, small variations in the distribution of trace elements. These features could have formed during multiple, separate diagenetic events, or they could represent a related continuum of products from a single event with heterogenous geochemical conditions and fluid transport pathways. Examination of crosscutting relationships of diagenetic features suggest at least one to three distinct episodes, so a combination of the above endmember scenarios may have occurred (Horgan, n.d.; L'Haridon et al., 2020). Sun et al. (2019) found the VRR‐forming members, Pettegrove Point and Jura, had smaller concretions than the rest of the Murray and suggested this observation demonstrated concretion formation postdated the initial cementation and loss of porosity in VRR. Uniquely constraining the number and styles of diagenetic episodes is not possible without detailed microanalysis, but several hypotheses are considered within the articles in this special issue (Bennett et al., 2018; Das et al., 2020; David et al., 2020; Frydenvang et al., 2020; Horgan, 2020; L'Haridon et al., 2020; McAdam et al., 2020; Rampe et al., 2020; Thompson et al., 2020; Wong et al., 2020).

#### 6.3.1 Geochemical Models

Gray hematite is defined by its coarser mineral grain size (>3–5 μm) compared to red hematite (Catling & Moore, 2003; Morris et al., 2020). We hypothesize the gray hematite patches represent localized zones of more thorough conversion of nanophase and fine‐grained red hematite into coarse‐grained gray hematite, associated with the widespread event that caused recrystallization and cementation across the ridge (Bennett et al., 2018; Horgan, 2020; Rampe et al., 2020). On Earth, gray hematite is most commonly found in hydrothermal settings (*T* = 100°C to 200°C) (Catling & Moore, 2003; Evenson et al., 2014; Jensen et al., 2018), and based on the arguments below, we propose that the gray hematite at VRR is evidence that the diagenetic fluids that altered VRR strata also had moderately elevated temperatures.

Fluids with even moderately elevated temperatures could have provided a route for accelerating Ostwald ripening across the ridge (Steefel & Van Cappellen, 1990). This mechanism has been suggested to explain the formation of iron oxide concretions and banding in the Navajo Sandstone (Potter et al., 2011; Wang et al., 2015), which experienced diagenetic temperatures of <100°C (Parry et al., 2004). Although the Navajo Sandstone has much lower abundances of iron than VRR (e.g., Beitler et al., 2005), this mechanism may generate localized hematite occurrences in otherwise bleached sandstone (Wang et al., 2015), potentially consistent with the centimeter‐scale bleaching patterns (Figure 9) and distribution of dark diagenetic features observed in VRR (Bennett et al., 2018). Fluids at elevated temperature may also have contained dissolved species capable of solubilizing Fe (III) via complexation (Scholten et al., 2019), which would accelerate the rate of coarsening.

The mineralogy of VRR somewhat constrains the temperature range permitted during a diagenetic event or events. The lack of conversion of feldspars to zeolites (Rampe et al., 2020) make a prolonged, regional thermal event unlikely because zeolites form subsequent to smectites during hydrothermal alteration and require Mg‐depleted, alkaline fluids (Alt, 1999). The lack of chlorite also suggests temperatures did not exceed ~200°C. Smectite in rocks of similar bulk composition as VRR are stable against conversion to chlorite up to temperatures between 100°C and 200°C, depending on the setting and fluid composition (Alt, 1999; Alt et al., 2010; Robinson et al., 2002). Brief thermal pulses, such as from transient hydrothermal fluids, are not observed to convert smectite to chlorite (Meunier, 2005). This is supported by hydrothermal alteration experiments, which found that smectites were the sole product of mafic rock alteration at 150°C after ~450 days of reaction (Seyfried & Bischoff, 1979). Conversely, the possible detection of ferripyrophyllite in VRR could indicate moderately elevated temperatures, but only if it is authigenic (McAdam et al., 2020; Rampe et al., 2020). One terrestrial occurrence of this mineral has an estimated formation temperature of ~60°C (Decarreau et al., 1990), with other reported occurrences associated with hydrothermal systems (Chukhrov et al., 1979), and no occurrences reported in sedimentary assemblages, suggesting elevated temperature is required for ferripyrophyllite formation.

Rampe et al. (2020) discuss possible heat sources that could have warmed diagenetic fluids in detail, which are summarized here. Diagenetic fluids with elevated temperatures may have been heated by burial and overburden of sediments combined with a greater past geothermal gradient. This may have resulted in temperatures up to 125°C or greater (Borlina et al., 2015). Diagenetic fluids may have also been warmed at depth by geothermal plumes, perhaps from magmatic activity infiltrating fractures in the crust created by the Gale crater impact and circulated for hundreds of meters to kilometers. Remnant heat from the Gale impact may have been a heat source too (Schwenzer et al., 2012). A final potential source of heat is radiogenic heat from the decay of unstable isotopes of K, U, and Th in the sediment. Notably, Gale crater sediments are enriched in K_2_O relative to average Mars crust (e.g., Bedford et al., 2019; Le Deit et al., 2016; Mangold et al., 2017; Siebach et al., 2017).

The formation of new phases during diagenesis is an integrated function of both temperature and time (Tosca & Knoll, 2009), so an alternative model to warm fluids is recrystallization and coarsening at cooler temperatures over long time periods. However, this model raises greater uncertainties. Gray hematite has not been reported in low‐temperature sedimentary environments to the best of our knowledge. In addition, current thermodynamic data predict that goethite, not hematite, is the stable iron oxide below 25°C to 60°C (Majzlan et al., 2003; Navrotsky et al., 2008). It is unclear whether hematite coarsening would occur during long aging times at cool temperatures when the thermodynamic driving force favors a different mineral. It is possible that preexisting hematite may coarsen over time via Ostwald ripening at low temperatures (Steefel & Van Cappellen, 1990), although localizing this phenomenon to the zone of gray hematite is difficult to explain why the gray hematite zone would have been exposed to cool fluids for an extended period of time.

Finally, L'Haridon et al. (2020) and David et al. (2020) explore an additional scenario, arguing that reducing conditions mobilized ferric phases in the diagenetic event that formed the Fe‐rich nodules (L'Haridon et al., 2020) or the entire ridge (David et al., 2020). Iron reduction provides a clear mechanism to deplete Fe in the halos around gray Fe‐rich overgrowths (Figure 9). However, the reducing species responsible for such a process is unclear. In terrestrial sedimentary units such bleaching is caused by hydrocarbon migration or H_2_S in brines, which may also lead to the formation magnetite or pyrite (Chan et al., 2000; Parry et al., 2004). While the dissolved Fe (II) generated can induce recrystallization of any remaining iron oxides, this preserves the original grain size and cannot drive coarsening (Frierdich et al., 2015; Handler et al., 2009, 2014; Rosso et al., 2010). Instead, preserving much of the iron content of the rock, and generating gray hematite, would also require introduction of an oxidant to convert any dissolved Fe (II) formed via reduction back into Fe (III).

#### 6.3.2 Fluid Pathways

The source of diagenetic fluids is not well constrained. Diagenetic fluids can originate from three sources: (1) connate water that is trapped by sediment as it is buried, (2) thermobaric water that is derived from hydrated minerals as sediments experience increased pressure and temperature, and (3) meteoric water that permeates the subsurface. Because of the evidence of late diagenesis, it is unlikely that connate water survived the burial and lithification of the Murray and overlying Stimson formations. Thermobaric water could have been derived locally from the conversion of smectite to ferripyrophyllite, opaline silica, and hematite (e.g., Rampe et al., 2020). Thermobaric water may have also been derived regionally from the dewatering of smectite‐bearing sediments as Mount Sharp formed. Meteoric water may have been present in the Gale crater subsurface for over a billion years after the deposition of the Murray formation. K‐Ar dating of jarosite in a sample from the Pahrump Hills demonstrated it formed 2.12 ± 0.36 Ga (Martin et al., 2017). The jarosite found by CheMin in VRR could not be dated by SAM, but the presence of hematite and jarosite in the Pahrump Hills and VRR may indicate formation during very late diagenesis. Although Rampe et al. (2020) hypothesize that the diagenetic fluids that influenced the mineralogy of VRR may have been sourced from a long distance as discussed below, there is evidence that some of these fluids were local and relatively static (e.g., halos surrounding gray Fe‐rich overgrowths; L'Haridon et al., 2020).

**Figure 17:**
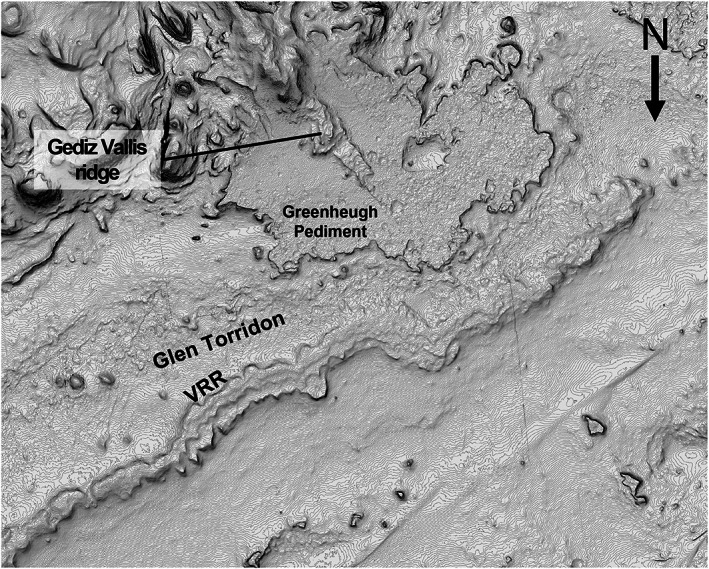
A 1 m contour map emphasizing topographic relationships between Vera Rubin ridge (VRR), Glen Torridon, and the Greenheugh pediment and unique shape of VRR.

The fluids responsible for enhanced diagenesis at VRR could have followed three pathways (Figure 16). In one scenario (Path 1), flows originating from precipitation, snowmelt, or dewatering of the overlying sulfate‐bearing strata may have been concentrated as surface runoff and shallow subsurface by Gediz Vallis, the canyon located directly south of VRR (Figure 1). Currently Gediz Vallis is downslope of an ~64 km^2^ catchment on the shoulder of Mount Sharp, and the arcuate shape of the diagenetically altered zone that resulted in the erosionally emergent ridge (Figure 17) may have been influence by localized fluids arriving from Gediz Vallis. Gediz Vallis ridge deposits appear to record water‐driven sediment transport down Gediz Vallis (Bryk et al., 2019; Palucis et al., 2016), further pointing to the flux of shallow water from Mount Sharp across the VRR location. At the base of Gediz Vallis, shallow subsurface fluids may have been further focused along the unconformity between the Greenheugh pediment and underlying Murray formation, taking advantage of changes in permeability and porosity associated with the contact. Topographic projections from the base of the modern‐day Greenheugh pediment suggest it could have once covered VRR, placing the top of VRR on what would have been a bounding surface of an unconformity (Bryk et al., 2019). After diagenesis, wind erosion cutting through the Greenheugh pediment cap may have etched out the southern side of an arcuate, diagenetically strengthened VRR deposit. This suggests that the hardening resulting from diagenesis found at VRR may not extend farther upslope. Although local groundwater flows likely occurred here, it is difficult to explain why fluids traveling along this pathway would have been warm.

Diagenesis could have alternatively been driven by deeper, regional subsurface flows traveling from the southern highland toward the northern lowlands through fractures in the Martian crust (Paths 2 and 3). Gale crater lies on the Martian dichotomy boundary and is notably downslope of the highlands to the south. The crater floor is the deepest point for hundreds of surrounding kilometers, and there is ample evidence of surface flows toward and probably into Gale (Irwin et al., 2005; Palucis et al., 2016). Irwin et al. (2005) traced a channel network originating on the rim of Herschel crater (about 600 km to the south) to Gale crater. Several smaller craters to the south of Gale were breached by channels entering from the south (Palucis et al., 2016). Horvath and Andrews‐Hanna (2017) report a hydrologic model of late stage lakes in Gale that links lake level to regional‐scale groundwater flow. Path 2 shows a shallower groundwater flow path that would cross through Mount Sharp. Path 3 shows a deeper path that would develop under a deep crater relative to a local or regional groundwater table. Hence, deep flows entering Gale could be forced up, possibly from great depths, and these waters may have been warmed from those depths. Future hydrological modeling could test the viability of either of these scenarios.

## 7 Conclusions

*Curiosity*'s exploration of Vera Rubin ridge addressed all three campaign goals:

1. *Campaign Goal 1:* Understand the primary depositional setting of the sedimentary rocks that make up the ridge and document their stratigraphic relationship with surrounding units. Rocks composing VRR were deposited in lacustrine and lacustrine margin settings with occasional intervals of subaqueous traction transport and are interpreted to be a continuation of the Murray formation (Edgar et al., 2020). Rocks within VRR are relatively flat lying and form a distinct geomorphic ridge feature due to postdepositional diagenetic processes rather than to variation in depositional processes and environments (Edgar et al., 2020; Stein et al., 2020).
2. *Campaign Goal 2:* Determine the source of the orbital hematite signature, understand its relationship with other hematite detections in Mount Sharp, and test the hypothesis that the hematite associated with the ridge indicated a site of past iron oxidation. The orbital spectral signature of hematite is caused by hematite associated with the bedrock of VRR. There is not a significantly greater abundance of hematite on VRR than surrounding rocks, but the ridge is associated with deeper spectral absorptions in situ that are likely due to enhanced crystallization or cementation caused by diagenesis (Fraeman et al., 2020; Horgan, 2020; Jacob et al., 2020; Rampe et al., 2020). The ridge does not preserve a redox interface.
3. *Campaign Goal 3:* Document additional primary and secondary geochemical environments that are preserved in the ridge. There is abundant textural, mineralogical, and chemical evidence of diagenetic overprinting across multiple spatial scales on VRR (Bennett et al., 2018; Das et al., 2020; David et al., 2020; Frydenvang et al., 2020; Horgan, 2020; L'Haridon et al., 2020; McAdam et al., 2020; Rampe et al., 2020; Thomas et al., 2020; Thompson et al., 2020; Turner et al., 2020; Wong et al., 2020). The ridge experienced a complex diagenetic history after sediments were initially deposited, and various hypotheses about geochemistry of diagenetic fluids are presented throughout this special issue (David et al., 2020; Frydenvang et al., 2020; L'Haridon et al., 2020; McAdam et al., 2020; Rampe et al., 2020; Turner et al., 2020; Wong et al., 2020).
4. *Curiosity*'s exploration of the ridge advances our knowledge of the history and habitability of Gale crater. The discovery that VRR itself was created by diagenetic processes provides a new example of the large‐scale effects of subsurface diagenesis on the Martian rock record. The continuation of predominantly lacustrine sedimentation recorded within the ridge demonstrates that habitable lakes persisted in Gale crater even longer than previously reported (Edgar et al., 2020; Grotzinger et al., 2015; Stack et al., 2019). While VRR does not represent a redox interface that would have marked a new kind of habitable environment, the evidence for at least one, and more likely multiple, late‐stage interactions with diagenetic fluids at VRR further expands the period of time when liquid waters would have been present at Gale crater, likely in the shallow or deep subsurface. The presence of coarse‐grained gray hematite on the ridge top in particular could indicate waters were warm and/or long lived, which could have provided promising environments in the shallow subsurface sheltered from surface radiation and temperature variations. Combined, these results suggest habitable environments at Gale crater may have been preserved late into the Hesperian, first at the surface and later in the subsurface.

## Data Availability

All of the data collected by *Curiosity* during the Vera Rubin ridge campaign can be found on the Planetary Data System (PDS, http://pds.nasa.gov), and most are easily accessible on the *Curiosity* Analyst's Notebook (https://an.rsl.wustl.edu/msl). This review contains no new data, and the reader should refer to the data statements in the cited manuscripts.
